# The impact of Semaphorin 4C/Plexin-B2 signaling on fear memory via remodeling of neuronal and synaptic morphology

**DOI:** 10.1038/s41380-019-0491-4

**Published:** 2019-08-23

**Authors:** Manuela Simonetti, Eszter Paldy, Christian Njoo, Kiran Kumar Bali, Thomas Worzfeld, Claudia Pitzer, Thomas Kuner, Stefan Offermanns, Daniela Mauceri, Rohini Kuner

**Affiliations:** 1 Institute of Pharmacology, Heidelberg University, Im Neuenheimer Feld 366, 69120 Heidelberg, Germany; 2 Institute of Pharmacology, Marburg University, Karl-von-Frisch-Str. 1, 35043 Marburg, Germany; 3 Department of Pharmacology, Max-Planck-Institute for Heart and Lung Research, Ludwigstrasse 43, 61231 Bad Nauheim, Germany; 4 Interdisciplinary Neurobehavioral Core, Heidelberg University, Im Neuenheimer Feld 515, 69120 Heidelberg, Germany; 5 Anatomy and Cell Biology Institute, Heidelberg University, Im Neuenheimer Feld 307, 69120 Heidelberg, Germany; 6 Department of Neurobiology, Heidelberg University, Im Neuenheimer Feld 366, 69120 Heidelberg, Germany

**Keywords:** Neuroscience, Psychiatric disorders, Physiology

## Abstract

Aberrant fear is a cornerstone of several psychiatric disorders. Consequently, there is large interest in elucidation of signaling mechanisms that link extracellular cues to changes in neuronal function and structure in brain pathways that are important in the generation and maintenance of fear memory and its behavioral expression. Members of the Plexin-B family of receptors for class 4 semaphorins play important roles in developmental plasticity of neurons, and their expression persists in some areas of the adult nervous system. Here, we aimed to elucidate the role of Semaphorin 4C (Sema4C) and its cognate receptor, Plexin-B2, in the expression of contextual and cued fear memory, setting a mechanistic focus on structural plasticity and exploration of contributing signaling pathways. We observed that Plexin-B2 and Sema4C are expressed in forebrain areas related to fear memory, such as the anterior cingulate cortex, amygdala and the hippocampus, and their expression is regulated by aversive stimuli that induce fear memory. By generating forebrain-specific Plexin-B2 knockout mice and analyzing fear-related behaviors, we demonstrate that Sema4C-PlexinB2 signaling plays a crucial functional role in the recent and remote recall of fear memory. Detailed neuronal morphological analyses revealed that Sema4C-PlexinB2 signaling largely mediates fear-induced structural plasticity by enhancing dendritic ramifications and modulating synaptic density in the adult hippocampus. Analyses on signaling-related mutant mice showed that these functions are mediated by PlexinB2-dependent RhoA activation. These results deliver important insights into the mechanistic understanding of maladaptive plasticity in fear circuits and have implications for novel therapeutic strategies against fear-related disorders.

## Introduction

Deregulated fear responses comprise a frequently occurring feature of several debilitating fear-related disorders, including post-traumatic stress disorder, and are characterized by an impaired ability to distinguish between danger and safety-related cues [1, 2]. Associating cues with aversive experience is a highly adaptive process that requires memory formation. It has been postulated that maladaptive plasticity in fear circuits can lead to persistent emotional responses in the lack of imminent danger, and form the basis of debilitating emotional disorders [3]. Therefore, understanding circuits and cellular and molecular mechanisms underlying behavioral abnormalities in fear responses is of prime importance towards developing effective therapies.

Rodent models of fear learning and memory employ the paradigms of fear conditioning to either an auditory-cued or a context to study memory formation and recall. Freezing responses recorded in rodents to auditory or contextual cues paired with aversive stimuli, such as foot shock, represent a form of associative learning that involves diverse forebrain areas, including the hippocampus, amygdala, prefrontal cortex and cingulate cortex [4, 5]. According to current knowledge, different stages of memory, namely acquisition, consolidation, recall and extinction involve specific brain areas and mechanisms and are operational over distinct time frames [6–8].

There is a large body of evidence for rapid, activity-dependent changes in synaptic transmission in all types of learning processes [9]. Furthermore, consolidation of memories not only involves functional changes in synaptic strength, but is also characterized by activity-dependent structural plasticity of synapses, such as changes in spine generation [10]. Spine remodeling in some brain areas has been reported in animal models of fear memory consolidation [11], but has not been extensively studied, and importantly, the molecular signaling processes that link neuronal activity to spine remodeling are not well-understood in the context of fear memory, unlike in spatial memory paradigms.

Key to spine remodeling in response to environmental stimuli are mechanisms which link signaling mechanisms at the cell surface to a rapid and discrete reorganization of the intracellular cytoskeletal elements [12, 13]. RhoGTPases, which are small intracellular molecular switches, are the most salient drivers of changes in the actin cytoskeleton. Indeed, members of the RhoA family of RhoGTPases are known to play important roles in morphogenesis of dendritic spines [14, 15] and synaptic plasticity [16–18] via cytoskeletal rearrangement [12] over development as well as in adult life. Although there is consensus that RhoGTPases sculpt dendritic and synaptic structure in response to sensory experience or during learning, much less is known about mechanisms that transmit environmental signals and influences to the activity of RhoGTPases.

During development, semaphorins, a large family of secreted and membrane-associated proteins, constitute one of the most important extracellular cues in axon navigation, branching, cell migration and specification. Class 3 semaphorins signal via Plexin-A family receptors and utilize neuropilins as co-receptors [19]. In contrast, several class 4 semaphorins bind and activate cell surface receptors of the Plexin-B-type. We and others have demonstrated that some class 4 semaphorins directly lead to the activation of neuronally expressed Rho-guanine exchange factors by virtue of direct protein-protein interactions between Plexin-B proteins and guanine exchange nucleotide factors (GEFs), such as PDZRhoGEF and LARG [20, 21]. Activation of the small GTPase, RhoA, and inhibition of other Rho-family GTPases, such as Rac and R-Ras, has been reported in response to Sema4–Plexin-B interactions in diverse cell types [20–22]. Amongst the known mammalian B-type plexins, Plexin-B1 and Plexin-B2 are expressed in developing neurons, whereas Plexin-B3 is mostly expressed in oligodendrocytes [23]. Plexin-B2 binds Semaphorin 4C (Sema4C) with high affinity, whereas Plexin-B1 only shows low affinity binding for Sema4C, binding Semaphorin 4D (Sema4D) with a high affinity instead [24]. Functional roles for both A- and B-type plexins and their semaphorin ligands have been reported in brain development, including synapse formation and maturation and regulation of dendritic morphology [25–27].

Although semaphorins were initially discovered as axon guidance cues in development [28], they are now emerging as modulators of adult life and disease pathogenesis in several organs and systems, such as tumor angiogenesis, growth and metastases, immune system regulation and pathological pain [29–34]. In the adult brain, some class 3 semaphorins have been linked to the regulation of synaptic strength and formation in mature neurons [35, 36]. However, potential functions of class 4 semaphorins and B-type plexins in adult brain have not been studied. Because their expression persists in the adult nervous system [34, 37], and particularly given that Plexin-B proteins have the ability to link environmental cues to the regulation of RhoGTPases, we addressed the potential regulation of expression and function of neuronal Plexin-B proteins in mechanisms involved in memory.

The specific aim of this study was to understand the impact of Sema4–PlexinB signaling on behaviors related to contextual and cued fear memory and address potential structural remodeling of basic units of memory, namely dendritic spines and synapses, in limbic circuits of the adult mouse brain. Our results from comprehensive experiments spanning expression analyses, diverse genetic models and behavioral and morphological studies indicate that Plexin B2 and its ligand, Sema4C, are critically involved in the expression of recent and remote fear memory, and bring about these functions via PlexinB2-dependent RhoA signaling by remodeling of the dendritic architecture and density of glutamatergic and GABAergic synapses in the hippocampus.

## Materials and methods

### Genetically modified mice

To generate mice lacking Plexin-B2 in forebrain-specific glutamatergic neurons, mice carrying a conditional allele for Plexin-B2 (PB2^fl/fl^) gene [24] were crossed with inducible CaMK-CreER^T2^ mice [38] induced twice per day i.p. with 50 mg/kg Tamoxifen over 5 consecutive days to obtain CaMK-CreER^T2+^, PB2^−/−^ mice—mice of this genotype following Tamoxifen administration are referred to as CaMK-PB2^−/−^ mice in this manuscript. Mice expressing the ß-galactosidase (*LacZ* gene) under the control of *plxnb2* and *sema4c* endogenous promoters (*Plexin-B2-LacZ* mice and *Sema4C-LacZ* mice, respectively) have been previously described [37, 39, 40] and previously validated in terms of faithful reproduction of expression patterns of the corresponding genes [34]. In both cases, the LacZ reporter is included in the targeted trap allele, and accordingly heterozygous knockout mice expressing LacZ were used here for expression analyses. Mice with a loss of RhoA function (LOF^RhoA^) were generated by expressing triple-myc-tagged wild-type Plexin-B2 or triple-myc-tagged Plexin-B2 (ΔVTDL) and have been described previously [41] (see Supplementary Fig. 1 for signaling pathway). Mice from the C57Bl6 background were used throughout the study. Mice were housed in the central facility of Heidelberg University in groups of 2–4 mice/group on a 12-h light–dark cycle with constant room temperature (RT). All animal use procedures were in accordance with ethical guidelines imposed by the local governing body (Regierungspräsidium Karlsruhe, Germany). All analyses were carried out by experimenters who were blinded to the genetic identity of mice or study groups, as appropriate.

### Analysis of ß-galactosidase activity and density

Mice were transcardially perfused with cold phosphate-buffered saline (PBS) followed by 0.5% cold paraformaldehyde (PFA). After a post fixation time of 6 h in 0.5% paraformaldehyde (PFA) at 4 °C, brains were incubated overnight in 30% sucrose in PBS at 4 °C. Brains were frozen and cryo-sectioned at 25 µm. Sections were kept in staining solution (0.1 M PBS pH 7.3, 2 mM MgCl_2_, 5 mM EGTA, 0.01% sodium deoxycholate, 0.02% NP-40, 10 mM potassium ferricyanide, 10 mM potassium ferrocyanide, 0.5 mg/ml X-gal) at 37 °C for 17 h in the dark. Brains of mock-treated mice were always processed in parallel with brains following fear conditioning (at day 3, 2 h after cue recall). Corresponding tissues from wild-type mice were always included and showed complete absence of signals.

For the density analysis, the lacZ images of rACC, amygdala and hippocampus were captured with an upright microscope (Nikon NiE, Nikon) equipped with Nikon plan Apo objective set and high-resolution CCD camera (Nikon DS-Ri1, Nikon). Each image was inverted, an area was defined and the mean density was calculated using the freely available software Fiji. For each condition, 4–5 images from at least 3 different mice were analyzed.

### Western blotting

The hippocampus, rACC and amygdala-enriched area of mouse brain were isolated from wild-type C57Bl6 mice that underwent fear conditioning or control C57Bl6 mice on day 3 (2 h after cue recall). The tissues were homogenized in ice-cold hypotonic buffer (25 mM Tris pH 7.4, 5 mM EDTA, 1 mM DTT) using a dounce homogenizer [18]. An equal volume of 2× ice-cold radioimmunoprecipitation buffer (600 mM NaCl, 100 mM Tris pH 7.4, 10 mM EDTA, 2% Triton X-100, 0.2% SDS, 1% sodium deoxycholate) was added to the tissues. The lysates were rotated for 30 min at 4 °C, centrifuged for 30 min at 4 °C, and supernatants were used for western blotting according to standard protocol.

Western blots were performed with the following antibodies: anti-Sema4C (sheep 1:200, PA5–47812, ThermoFisher Scientific), anti-Plexin-B2 (sheep 1:700, PA5-47880, Thermo Fisher Scientific). An antibody recognizing ß-tubulin III (rabbit 1:6000, T2200, Sigma Aldrich) was used to quantitate ß-tubulin III expressed constitutively in neurons as a loading control. Membranes were incubated with anti-rabbit (1:6000), or anti-sheep (1:4000) HRP-conjugated antibodies and developed using Amersham™ ECL™ (GE Healthcare) and Hyperfilm MP (Amersham). At least four samples from independent experiments were analyzed by densitometry using Fiji and corrected for loading by normalization to corresponding bands for ß-tubulin III.

### RNAscope ISH assay

Fresh frozen brain slices were analyzed with RNAscope assay (Advanced Cell Diagnostics, Hayward, CA, USA) using target probes for *Plexin-B2* (RNAscope® Probe—Mm-Plxnb2 459181, ACD) and *Sema4C* (RNAscope® Probe—Mm-Sema4c-C3 # 518631-C3, ACD), following the manufacturer’s instructions. Briefly, mice were perfused with cold PBS, the brain was quickly dissected, immersed in OCT and frozen in dry ice. Frozen brains were stored at −80 °C. The brains were then sectioned with a cryostat at 20 µm thickness and sections were stored at −80 °C.

Slides were removed from −80 °C and immediately immersed in 4% PFA in PBS for 20 min at 4 °C after fixation, rinsed three times with 1 PBS and tissues were dehydrated in 50%, 70 and 100% EtOH for 5 min each at RT. The sections were stored overnight at −20 °C in 100% EtOH. Slides were that incubated with the solution Pretreat 4 (source: ACD) for 30 min at RT. After washing, the slides were hybridized with probes against *Sema4c* and *Plxnb2* for 2 h at 40 °C using HybEZ™ Oven; probes were 20 bps each, and the target regions were located between 1750 and 2682 bp of the murine *Sema4c* gene and between 1207 and 2153 bp of the *Plxnb2* gene, respectively (precise sequence being proprietary information of ACD). Signal amplification was performed using the RNAscope® Fluorescent Multiplex Reagent Kit (ADC).

The slides were hybridized at 40 °C with Amp 1-FL for 30 min, with Amp 2-FL for 15 min, with Amp 3-FL for 30 min and with Amp4-FL for 15 min. Between each step, the slides were washed twice with washing buffer (ACD) for 2 min. We chose the Amp4-FL-Alt A option with C1 and C3 target probes in Green and Far Red. The sections were counterstained with DAPI (ACD) for 30 s at RT.

RNAscope results were analyzed with Leica SP8 confocal microscope taking images with ×63 objective (further two-fold magnification). For each condition, we analyzed at least 3 different images per section, 3 sections per mouse, and 3 mice per condition. Using Fiji software, we measured the integral signal intensity for each probe and normalized it to the number of cell nuclei counterstained with DAPI. With the same software, we also analyzed the mean number of puncta per nucleus by counting the total number of puncta and normalizing to the number of cell nuclei (DAPI staining).

### Immunofluorescence staining on brain sections

Mice were transcardially perfused with cold PBS and quickly decapitated. The brains were rapidly extracted, rinsed in ice-cold PBS, fixed in ice-cold 4% paraformaldehyde (PFA) for 30 min, cryopreserved in 30% sucrose overnight, and cryo-sectioned at 25 µm. Following Na-Citrate antigen retrieval treatment, the hippocampal brain sections were stained with rabbit anti-PSD-95 (1:200, Invitrogen), mouse anti-Gephyrin (1:500, SYSY), or mouse anti-pan-Homer1 (1:200, Santa Cruz) antibodies using standard protocols for immunofluorescence staining [42]. The sections were analyzed using a confocal laser-scanning microscope (Leica TCS SP8).

### Analysis of synaptic density in brain sections

For analyses, only synapses located at the CA1 region of the hippocampus were chosen. For each condition, three different ROIs were analyzed. The imaged ROIs were randomly chosen in the stratum radiatum of the hippocampal CA1 area. *Z*-stack images spanning 5 µm on *Z*-axis (10 images, one every 0.5 µm) were captured using a confocal laser-scanning microscope (TCS SP8, Leica), and analyzed with Fiji software. After 3D reconstruction, the same threshold was applied on each 3D image followed by processing with the 3D object counter tool. The data are shown as number of anti-PSD-95-, anti-gephyrin- or anti-pan-Homer1-immunoreactive puncta per µm^3^ × 100.

For the analysis of PSD-95 puncta size at least 10 puncta were randomly choose in each image. Images were processed by using a threshold at a constant level, yielding a binary image (mask) containing a discrete number of clusters (binary value 1) over background (binary value 0). By applying a threshold, a discrete border was created around objects. Using the function “analyze particles” of Fiji software automatically yielded a series of parameters describing each identified particle, such as area and perimeter. Since we could identify bigger signal clusters in which we were not able to identify and analyze separately the single PSD-95 puncta both with the software or manually, we arbitrary set a cutoff of 0.3 µm^2^. For each condition, we analyzed at least 3 images per mouse from 3 mice per group.

### qRT-PCR

CA1 tissue was dissected in RNAlater and rapidly frozen. Total RNA was extracted using the RNeasy MiniKit (Qiagen) including an optional DNase I treatment at RT for 15 min according to manufacturer’s instructions (Qiagen). Extracted RNA was reverse transcribed into first strand cDNA using High Capacity cDNA Reverse Transcription kit (Applied Biosystems). Quantitative reverse transcriptase PCR (qRT-PCR) was done on a StepOne plus Real Time PCR system using TaqMan Gene Expression Assays for the indicated genes (Applied Biosystems). The following TaqMan Gene Expression Assay was used in this study: *Arc* (Mm00479619_g1). Expression of target genes was normalized against the expression of *Gusb* (Mm00446953_m1), which was used as an endogenous control gene. For each condition, seven mice were tested (*n* = 7 mice).

### Elevated plus maze

Elevated plus maze was used to determine anxiety levels. Mice were placed in the center of the elevated plus maze and were allowed to freely move in the maze for 10 min. The behavior of the mice was videotaped and analyzed [43].

### Fear conditioning

All studies took place during the light period cycle. Mice were handled gently for 2–5 min for 3 days. The procedures used for contextual fear conditioning and cued fear conditioning were similar to those described previously [44, 45]. Briefly, on day 1, mice were placed in a fear-conditioning chamber for total of 360 s. A 5000 Hz (85 dB) tone lasting 30 s was delivered four times and was followed by a 1-s foot shock (0.6 mA). For contextual fear retention, the mice were returned to the same chamber on day 2 (recent memory) and on day 36 (remote memory) and allowed to move freely for a total of 360 s. No tone or foot shocks were delivered. Freezing behavior was recorded and analyzed. For auditory-cued fear retention, mice were placed in a novel chamber on day 3 (recent memory) and on day 37 (remote memory) and allowed to move freely for a total of 240 s. Two tones (5000 Hz, 85 dB) lasting for 30 s were delivered. Freezing behavior was recorded and analyzed. Control mice were handled in a similar manner, and placed in the same chambers as the test mice with the difference that no foot shock was applied (mock controls).

### Golgi–Cox staining and tissue preparation

Golgi–Cox staining was used to determine post-conditioning changes in neuronal morphology at the recent memory (day 3) and remote memory (day 37) time points, and was performed by using a FD Rapid Golgi Stain Kit (FD Neurotechnologies [46]). Briefly, on day 3 and on day 37, mice were killed and brains were dissected and impregnated (equal volumes of Solutions A and B, containing mercuric chloride, potassium dichromate, and potassium chromate) and stored at RT. Impregnation solution was replaced after 24 h. After 15 days, the brains were transferred to Solution C and stored at 4 °C for 48 h, replacing solution at 24 h. The brain was cryo-sectioned sagittally (100μm). The sections were mounted on gelatin-coated microscope slides with Solution C and were stained according to the manufacturer’s protocol.

### Quantitative analyses of neuronal morphology in brain sections

Spine density was measured on pyramidal neurons located in the CA1 region of the dorsal hippocampus. Neurons, identified with a Nikon Ni-E widefield microscope under low magnification (×10), were chosen by locating the regions of interest. Neurons showing at least third-order branches for both apical and basal dendrites were selected. In total, 12 neurons were studied per each group. Only those dendrites were chosen for analysis which met the following criteria: (1) presence of intact dendrites, (2) consistent and dark impregnation along the neuron, (3) pertinent isolation from neighboring impregnated neurons. Afterwards, dendritic spines were analyzed under higher magnification (×63) with a Nikon upright microscope (Nikon NiE) equipped with Nikon plan Apo objective set and high-resolution CCD camera (Nikon DS-Ri1, Nikon) and acquisition process was controlled by NIS-Element software 4.1 (Nikon).

For dendrite analyses purposes, only impregnated neurons that are located at the CA1 region of the hippocampus were chosen. In addition, the dendrite of the chosen neuron must be well separated and distinguishable from dendrites of neighboring neurons to avoid mistracing. *Z*-stack images of these neurons were captured with using an upright microscope (Nikon NiE, Nikon) equipped with Nikon plan Apo objective set and high-resolution CCD camera (Nikon DS-Ri1, Nikon) and acquisition process was controlled by NIS-Element software 4.1 (Nikon). The stack-image of each neuron was inverted, manually traced and filled using Fiji and its Simple Neurite Tracer plug-in to create a representative traced image of the neuron in binary mode. The .swc files created during the tracing process were further used for the dendrite quantification (length and numbers) and the Sholl analysis was performed with 5 µm shell interval by using the centre of the soma as reference point [47] as described previously [48].

### Hippocampal primary culture and transfection

Hippocampal neurons from newborn C57BL/6 mice were cultured as described [49, 50]. DNA transfection with a plasmid encoding for hrGFP was performed after a culturing period of 8 days in vitro (DIV) using Lipofectamine 2000 (Invitrogen). Experiments were done at DIV10. Semaphorin 4C (150 nM), Y-27632 (3.3 µM) and PHA 665752 (2.5 µM) were applied for 24 h, with a pre-incubation time for Y-27632 and PHA 665752 of 30 min.

### Image acquisition and morphometric analyses

A confocal laser-scanning microscope (TCS SP2, Leica, Mannheim, Germany) equipped with an inverted fluorescence microscope (DM IRE2, Leica) and Leica confocal scan software was used to acquire fluorescence images of the hippocampal primary cultured neurons. In order to perform morphometric analyses of dendrites and spines, all images were acquired with a sequential setting and a resolution of 1024 × 1024 pixels. Each image is composed by a *z*-series projection of pictures taken at 1 µm depth intervals for dendrites and 0.5 µm depth intervals for spines.

Sholl analysis was applied to investigate total dendritic length and spine density using the freely available software Fiji. Briefly, *z*-stack images of neurons were captured imported in Fiji and manually traced using the simple neurite tracer plug-in. The representative traced image of the neuron was used for the quantification of total dendrite length. The Sholl analysis was performed with 5 µm shell interval. For the analysis of dendritic spine density, 20 µm of dendrite portions were randomly chosen and manually computed. All analyses were performed in blind. For each condition, 11–12 neurons from 3 independent preparations were analyzed in each experiment.

### RhoA activation assay

A luminescence-based G-LISA™ RhoA activation assay kit (Kit # BK121, Cytoskeleton, Inc., Denver, CO) was used to determine RhoA activity according to the manufacturer’s instructions. Briefly, cultured hippocampal neurons were exposed to 150 mM recombinant Sema4C, and subsequently washed with ice-cold PBS. Cells were lysed and total proteins were harvested. The protein concentration was determined according to manufacturer’s instructions and cell extracts were equalized to a protein concentration of 1.5 mg/ml for assay. After incubating for 45 min at RT with the primary anti-RhoA antibody, luminescence intensity was measured at 490 nm in Luminoskan™ Ascent Microplate Luminometer (5300173, Thermo Scientific, USA), according to the manufacturer’s recommendation.

### Statistics

Student’s *t*-test was employed while comparing two genetic groups with each other for a single parameter and single time point. In RNAScope analyses, differences between the two groups tested were compared using an unpaired *t*‐test. Analysis of variance (ANOVA) for random measures was employed in experiments comparing multiple groups or multiple time points and post hoc Bonferroni’s or Tukey’s test for multiple comparisons was performed, as indicated. In qPCR analyses, ANOVA with Holm–Sidak correction for multiple comparisons was employed. For non-parametric data in the analysis of bouton size, Krusker–Wallis test was employed. *N* numbers of all data sets are provided in figure legends. All data in the figures are expressed as mean ± SEM.

## Results

### Analysis of expression of Plexin-B2 in brain regions involved in fear memory

**Fig. 1:**
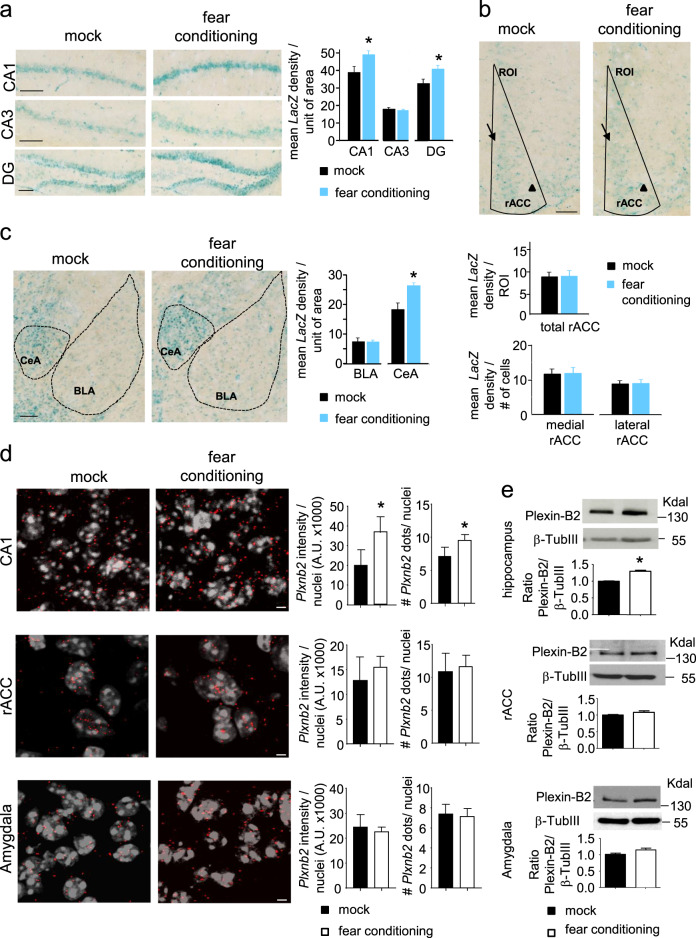
Expression patterns and regulation of *Plxnb2* mRNA and Plexin-B2 protein in brain regions involved in fear and fear memory. **a**–**c** Analysis of β-galactosidase expression in mice expressing the *LacZ* gene in the *Plxnb2* locus (Plexin-B2-LacZ mice) across the CA1, CA3, and DG areas of hippocampus (**a**), anterior cingulate cortex (rACC; **b**) and in the basolateral amygdala (BLA) and central amygdaloid nucleus (CeA; **c**). Typical examples, and quantitative summary of densitometric magnitude of LacZ staining in mock-treated mice and at day 3 following fear conditioning via foot shock stimulation. In panel **b**, bar graphs represent mean signal density over unit area over the whole ROI (demarcated by black lines in the examples shown in panel **b**) as well as a separate analysis of LacZ staining signal intensity in the high and low expression level areas (medial and lateral areas, respectively; indicated by arrow and arrowhead, respectively), normalized on the number of cells present in the ROI; *n* = 3 mice/group; at least 3 sections were analyzed per mouse. **d** Analysis of *Plxnb2* mRNA expression across the CA1 area of hippocampus (upper panels), rostral anterior cingulate cortex (rACC; middle panels) and in the central amygdala (CeA; lower panels) using RNAScope *in situ* hybridization (ISH) technique. Typical examples (images) and quantitative summary (bar graphs) of signals achieved with the *Plxnb2* probe in mock-treated mice and mice with fear conditioning at day 3 following foot-shock; bar graphs on the left represent the integral signal intensity normalized on the number of cell nuclei, whereas bar graphs on the right represent the mean number of signal dots per nucleus; *n* = 3 mice per group; at least 3 sections were analyzed per mouse. **e** Examples and densitometric quantification of signals in Western blot analysis of Plexin-B2 protein expression in lysates of CA1 area of hippocampus (upper panels), rACC (middle panels) and the amygdalae-enriched tissue (lower panels) in mock-treated mice and following fear conditioning at day 3 following foot-shock; data are represented as fold changes of the ratio of Plexin-B2 levels over loading control signal (β-Tubulin III); *n* = 4 mice per group. Student’s *t*-test. *P* < 0.05 indicated by * as compared to the corresponding control (mock) group. Error bars represent SEM. Scale bars represent 100 µm (**a**–**c**) and 5 µm (**d**)

We commenced this study by analyzing the expression of the gene encoding Plexin-B2, namely *plxnb2*, over brain areas involved in fear memory and studying potential changes in paradigms of fear memory recall. Owing to limitations of commercially-available anti-Plexin-B2 antibodies for immunohistochemistry, we utilized mice expressing the highly sensitive and reliably detectable reporter, β-galactosidase (*LacZ gene)* under the control of the *plxnb2* promoter (*Plexin-B2-LacZ* mice) [40], to map the expression of *plxnb2* in adult forebrain. We have previously demonstrated that *Plexin-B2-LacZ* mice faithfully represent the expression of the *plxnb2* gene, both in pattern and induction levels [34]. In order to quantitatively assess the intensity of LacZ staining, all samples were stained, processed, imaged and analyzed together. We observed expression of β-galactosidase in area CA1, area CA3 and the dentate gyrus (DG) of the hippocampus (Fig. 1a) and in other regions involved in fear memory, such as the rostral anterior cingulate cortex (rACC) (Fig. 1b) and the amygdaloid nuclei (Fig. 1c). Within 1 day following fear conditioning in *Plexin-B2-LacZ* mice, we observed that β-galactosidase expression is increased in the DG and the CA1, but not in the CA3 as compared to mock-treated *Plexin-B2-LacZ* mice (typical examples on the left, densitometric quantification in the bar graphs on the right in Fig. 1a). We observed differential β-galactosidase expression in the medial (arrowheads in Fig. 1b) versus the lateral aspects of the rACC; however, no differences were observed between *Plexin-B2-LacZ* mice with mock treatment or fear conditioning (typical examples and quantitative summary in Fig. 1b; Supplementary Fig. 2). In *Plexin-B2-LacZ* mice, LacZ expression was observed in both basolateral amygdala (BLA) as well as the central amygdaloid nucleus (CeA), of which, only the CeA showed an increase in magnitude of expression at day 1 after fear conditioning (typical examples and quantitative summary in Fig. 1c). This suggests that the *Plxnb2* gene is expressed in the forebrain, including some regions linked to fear memory, and its expression is further increased in some areas upon fear conditioning.

This was further tested via two additional, independent methods, namely RNAscope-based mRNA in situ hybridization and western blotting. In RNAscope experiments, *Plxnb2* mRNA expression was observed in the hippocampus, amygdala and rACC (Fig. 1d and Supplementary Fig. 3b; negative controls shown in Supplementary Fig. 3) and to study potential regulation, the intensity of mRNA signals (red dots) was normalized to the intensity of nuclear staining (white signals) within each image. Quantitative densitometric analyses showed an upregulation of *Plxnb2* mRNA expression in the CA1, but not in the rACC or central amygdala in mice at 24 h after fear conditioning (Fig. 1d). Finally, western blots demonstrated a significant increase in Plexin-B2 protein expression in the hippocampus, but not in the rACC or the amygdaloid complex at 24 h after fear conditioning (Fig. 1e). We have previously reported on the validation of the Plexin-B2 antibody employed here for its specifity in western blotting [34]. These results provide converging evidence that Plexin-B2 is upregulated in area CA1 of the hippocampus within a day upon fear conditioning.

### Analysis of expression of Sema4C in brain regions involved in fear memory

**Fig. 2:**
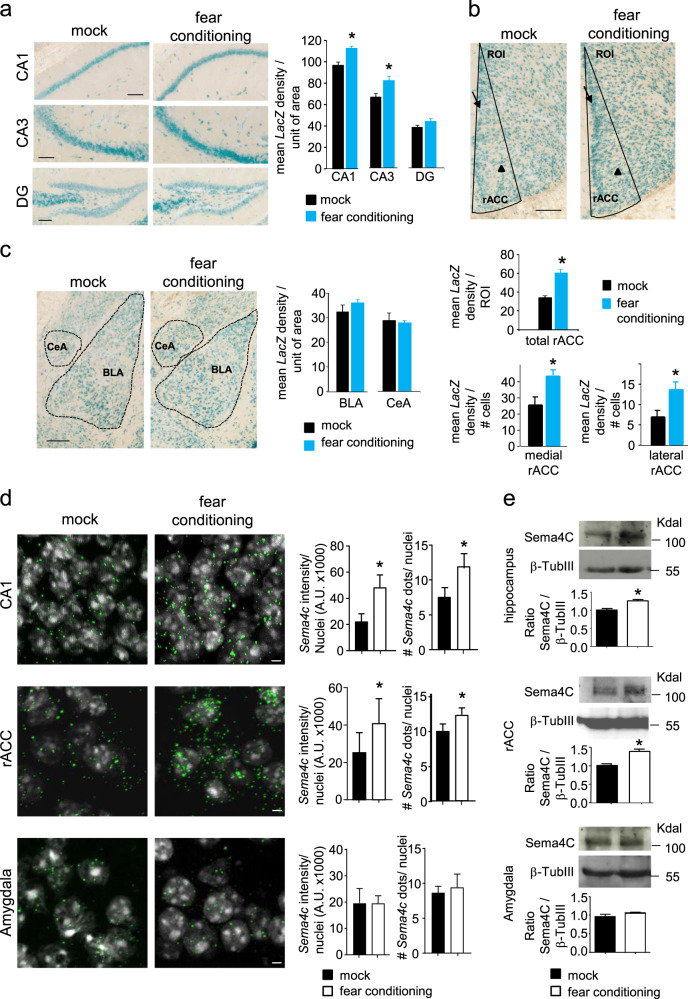
Expression and regulation of *Sema4c* mRNA and Sema4C protein in basal and fear memory conditions. **a**–**c** Analysis of β-galactosidase expression in mice expressing the *lacZ* gene in the *Sema4c* locus (Sema4C-LacZ mice) across the CA1, CA3, and dentate gyrus (DG) areas of hippocampus (**a**), anterior cingulate cortex (rACC; **b**) and basolateral amygdala (BLA) and central amygdaloid nucleus (CeA; **c**). Typical examples and quantitative summary of densitometric magnitude of LacZ staining in mock-treated mice and at day 3 following fear conditioning with foot shock. In panel b, bar graphs represent mean signal density over unit area over the whole ROI (demarcated by black lines in the examples shown in panel **b**) as well as a separate analysis of LacZ staining signal intensity in the high and low expression level areas (medial and lateral areas, respectively; indicated by arrow and arrowhead, respectively), normalized on the number of cells present in the ROI *n* = 3 mice per group; at least 3 sections were analyzed per mouse. **d** Analysis of *Sema4c* mRNA expression across the CA1 area of hippocampus (upper panels), rACC (middle panels) and in the central amygdala (lower panels) using RNAScope ISH technique. Typical examples (left), and quantitative summary (bar graphs) of *Sema4c* probe signal in mock-treated mice and on day 3 following fear conditioning upon foot shock; bar graphs on the left represent the integral signal intensity normalized on the number of cell nuclei, whereas bar graphs on the right represent the mean number of signal dots per nucleus; *n* = 3 mice/group; at least 3 sections were analyzed per mouse. **e** Examples and densitometric quantification of Sema4C protein via western blot analysis in lysates of CA1 area of hippocampus (upper panels), rACC (middle panels) and amygdalae-enriched tissue (lower panels) in mock-treated mice and on day 3 following fear conditioning via foot shock; data are represented as fold changes of the ratio of Sema4C over loading control signal (β-Tubulin III); *n* = 4 mice per group. Student’s *t*-test was performed; *P* < 0.05 indicated by * as compared to the corresponding control (mock-treated) groups. Error bars represent SEM. Scale bars represent 100 µm (**a**, **c**) and 5 µm (**d**)

Sema4C is a high-affinity ligand for Plexin-B2 [51]. We therefore analyzed the expression of Sema4C over brain areas involved in fear memory and studied potential changes in paradigms of fear memory recall. Owing to limitations of commercially available anti-Sema4C antibodies for immunohistochemistry, we utilized a knock in Sema4C reporter mouse line [40] (*Sema4C-LacZ*). In a previous study, we have demonstrated that in this mouse line, expression of the *LacZ* gene, which is knocked into the *Sema4c* gene locus, faithfully recapitulates the expression as well as induction of the *Sema4c* gene [34]. We observed expression of β-galactosidase in the hippocampal CA1- and CA3 regions, which was significantly enhanced within 24 h after fear conditioning (Fig. 2a). *LacZ* expression was also found in the DG, which remained unchanged upon fear conditioning (Fig. 2a). *LacZ* expression was also evident in the rACC, where its showed significant increase in mice at 24 h after fear conditioning (Fig. 2b; shown are density values across the whole rACC, as well divided between high expression zone (medial, indicated by arrow) and low expression zone (lateral, indicated by arrowhead; see also Supplementary Fig. 2b, c). LacZ staining was also detectable in both the basolateral amygdala (BLA) as well as the central amygdaloid nucleus (CeA) in Sema4C-LacZ mice, which remained unchanged in intensity at 24 h after fear conditioning (Fig. 2c). Similarly, we also performed RNAscope in situ hybridization experiments on *Sema4c* mRNA (Fig. 2d) and western blot analyses (Fig. 2e) with respect to Sema4C protein expression. Importantly, they completely confirmed the observations made with Sema4C-LacZ reporter mice, namely that both mRNA as well as protein are significantly enhanced in CA1 and rACC, but not in the amygdala upon fear conditioning.

### Plexin-B2 and its ligand Sema4C, but not Plexin-B1, are functionally involved in fear memory

**Fig. 3:**
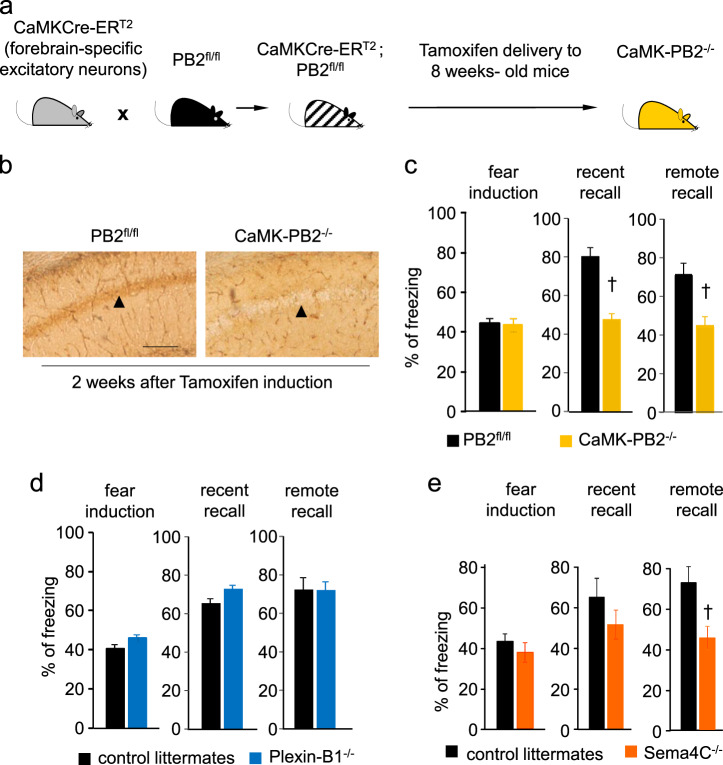
Analysis of function of Plexin-B2 in brain regions involved in fear and fear memory. **a** Schematic representation of strategy for generation of mice lacking *plxnb2* conditionally in forebrain excitatory neurons in a Tamoxifen-inducible manner. **b** Immunohistochemistry demonstrating loss of Plexin-B2 expression in CA1 neurons on hippocampus 2 weeks post-Tamoxifen administration to adult CaMK-PB2^−/−^ mice. **c**–**e** Analysis of contextual fear memory over recent (2 days) and remote (36 days) stages post-fear conditioning in CaMK-PB2^−/−^ mice and their control littermates (PB2^fl/fl^, **c**; *n* = 8 mice per group), or in mice constitutively lacking *Plxnb1* (Plexin-B1^−/−^ mice) and their control wild-type littermates (**d**; *n* = 8 mice per group), or in mice globally lacking *Sema4c* (Sema4C^−/−^ mice) and their control wild-type littermates (**e**; *n* = 7 mice per group) Student’s *t*-test. *P* < 0.05 indicated by ^†^ as compared to the corresponding control (wild-type) littermates. Error bars represent SEM. Scale bars represent 500 µm (**b**)

We then tested Plexin-B2 function in the context of fear memory, seeking systems to conditionally delete the *plxnb2* gene in areas that are relevant to fear memory in a temporally-inducible manner, in order to circumvent developmental defects arising from constitutive gene deletion of *plxnb2* [24] as well as any adaptive changes. Mice carrying floxed alleles of the *plxnb2* gene (PB2^fl/fl^ mice [24, 41]) were crossed with CaMKCreER^T2^ mice [38], which enable gene deletion in a forebrain-specific and Tamoxifen-inducible manner to generate forebrain-specific inducible Plexin-B2 knockout mice (CaMK-PB2^−/−^) (Fig. 3a). Administration of Tamoxifen at 50 mg/kg twice a day i.p. over 5 consecutive days at 8 weeks of age led to a loss of Plexin-B2 in forebrain neurons tested via immunohistochemistry 2 weeks later in the brains of CaMK-PB2^−/−^ mice, but not control PB2^fl/fl^ mice (Fig. 3b).

At 12 weeks of age, we tested CaMK-PB2^−/−^ mice and their control PB2^fl/fl^ littermates for freezing behavior in classical fear conditioning paradigms, as described previously [44, 45] (Supplementary Fig. 1b). Fear induction, i.e., freezing response during shock, was comparable in CaMK-PB2^−/−^ mice and PB2^fl/fl^ littermates (Fig. 3c). In contextual fear tests, the magnitude of recent memory (tested at 2 days after fear conditioning) as well as remote memory (tested at 36 days) was significantly reduced in CaMK-PB2^−/−^ mice as compared to PB2^fl/fl^ mice (Fig. 3c).

Plexin-B1 is a high affinity receptor for Sema4D and a low affinity receptor for Sema4C [24] and Sema4A [24, 52]. Although it is not well-expressed in adult mouse brain, a weak expression has been reported in adult mouse hippocampus [53]. Our previous detailed characterization of mice lacking *plxnb1* in a constitutive and global manner (Plexin-B1^−/−^) showed that they do not have any developmental abnormalities in the nervous system [24]. In the fear memory tests described above, Plexin-B1^−/−^ mice showed comparable behavior to their corresponding control littermates in both recent and remote contextual fear memory recall (Fig. 3d). Thus, amongst the neuronal plexin-B family members, only Plexin-B2 plays a role in contextual fear memory formation and recall.

To assess whether Sema4C functionally affects fear induction or recall, we employed mice globally lacking the *sema4c* gene (Sema4C^−/−^ mice), given especially the unavailability of mice with floxed alleles. We observed that while fear induction occurred normally in Sema4C^−/−^ mice, there was a trend for reduced recent recall and a significant drop in the magnitude of the remote recall in contextual memory tasks (Fig. 3e).

### Fear conditioning enhances dendritic complexity and spine density in hippocampal CA1 pyramidal neurons of wild-type mice, but not in mice lacking Plexin-B2

The hippocampus is one of the regions of the mammalian brain that shows high capacity for structural reorganization, which is closely associated with learning and memory processes, including fear memory [44]. We therefore analyzed changes at spine level as well as dendritic arbors in dorsal hippocampal neurons as potential neurobiological mechanisms to account for the observed impairment of memory induction and recall in CaMK-PB2^−/−^ mice. We focused on CA1 neurons given the strong expression and induction of the *LacZ* gene in Plexin-B2-LacZ mice.

**Fig. 4:**
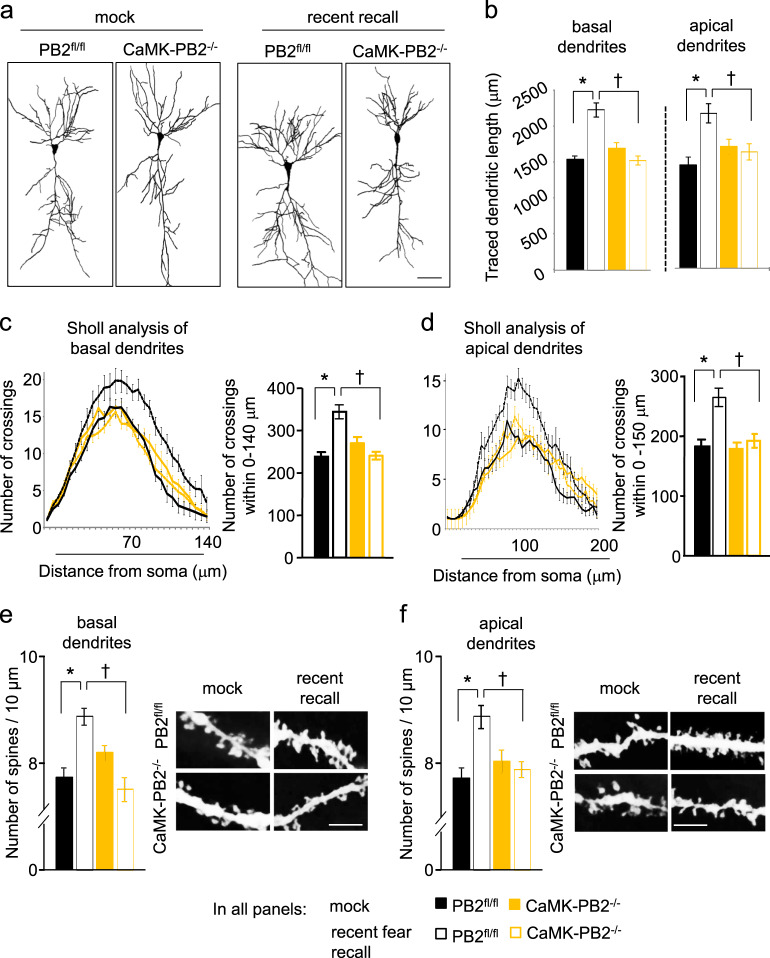
Role of Plexin-B2 in fear memory-related remodeling of dendritic complexity and structural plasticity of dendritic spines in hippocampal neurons in vivo. **a**, **b** Typical examples of changes in the morphology of Golgi-stained CA1 neurons (**a**) and quantitative analysis (**b**) of total length of apical or basal dendrites in CaMK-PB2^−/−^ mice and PB2^fl/fl^ littermates in basal state or at 3 days after fear conditioning (corresponding to recent recall). **c**, **d** Cumulative frequency plot of Sholl analysis (left panel) and plot of the average number of dendritic crossings (right panel) in defined distance from the soma of basal (**c**) and apical (**d**) dendrites in the treatment groups described above. **e**, **f** Typical examples of Golgi-stained dendritic segments (right panel) and quantitative analysis (left panel) of dendritic spine density over basal (**e**) and apical (**f**) dendrites of CA1 pyramidal neurons in the treatment groups described above. In all panels, a minimum of 12 neurons per genotype or treatment from at least 3 mice were analyzed. Two-way ANOVA followed by Bonferroni’s test was performed. * represents *P* *<* 0.05 when mice with fear conditioning and corresponding mock-treated mice were compared and ^†^ represents *P* *<* 0.05 when mutant and corresponding wild-type littermates were tested. Error bars represent SEM. Scale bars represent 50 µm (**a**) and 5 µm (**e**, **f**)

We used Golgi staining to assess changes in neuronal morphology in separate sets of mice that were tested before and after induction of fear memory, both over recent and remote memory time points, employing widely-accepted methods for quantifying dendritic spines and dendritic ramification (see ‘Methods’ for details). A typical example of Golgi-stained CA1 hippocampal neuron before and after image processing is shown in Supplementary Fig. 4a. We found that under basal conditions, CA1 neurons from CaMK-PB2^−/−^ mice showed no significant differences to those from control mice (Fig. 4a, b). Upon studying neuronal morphology of CA1 neurons in Sholl analyses, we observed that the complexity of the dendritic tree was also comparable between naïve CaMK-PB2^−/−^ mice and their control littermates (Fig. 4c, d). We then studied how dendritic length and arborization of CA1 neurons are affected by spatial fear memory induction in adult wild type and whether Plexin-B2 potentially plays a role. While control mice showed a significant increase in the total length of both apical and basal dendrites at day 2 after fear conditioning, i.e., corresponding to stages of recent fear memory recall, CaMK-PB2^−/−^ mice did not (Fig. 4a, b). Similarly, in Sholl analyses, control mice, but not CaMK-PB2^−/−^ mice, showed a significant increase in the number of dendritic crossings in both apical and basal dendrites of CA1 neurons at the time of recent fear memory recall (Fig. 4c, d).

Diverse forms of learning and memory are closely linked to synaptic plasticity [54–56]. Structural plasticity of hippocampal synapses has been reported in some previous studies in response to contextual fear memory training [52, 57–59]. We therefore tested how this is modified by lack of Plexin-B2 in CaMK-PB2^−/−^ mice. Quantifying spine-like dendritic protrusions in Golgi-stained CA1 neurons revealed that under basal conditions, spine density in basal and apical branches was comparable in Golgi-stained CA1 neurons in CaMK-PB2^−/−^ mice and control mice (Fig. 4e, f). At time points corresponding to recent fear memory recall, the spine number was markedly increased over basal value at both apical as well as basal dendrites of CA1 neurons in the control mice (Fig. 4e, f) in a magnitude comparable to previous reports [57, 58]. Importantly, fear memory-related increase in spine density did not occur in CaMK-PB2^−/−^ mice (Fig. 4e, f). In contrast to recent memory-related changes, at the time of recall of remote fear memory, spine number in hippocampal CA1 neurons was not significantly altered at in either wild-type mice or CaMK-PB2^−/−^ mice as compared to basal conditions (Supplementary Fig. 4b). This is consistent with previous publications reporting that spine density in remote memory stages is altered in ACC, but not in CA1 [11]. Thus, the dendritic arborization as well as density of dendritic spines in CA1 neurons are increased in complexity upon acquisition of fear memory and present a structural correlate of functional changes leading to memory recall in wild-type mice, but not in mice lacking Plexin-B2 in adult forebrain neurons.

### Fear memory-related alterations in overall density of excitatory and inhibitory synapses in the stratum radiatum zone in hippocampal CA1

**Fig. 5:**
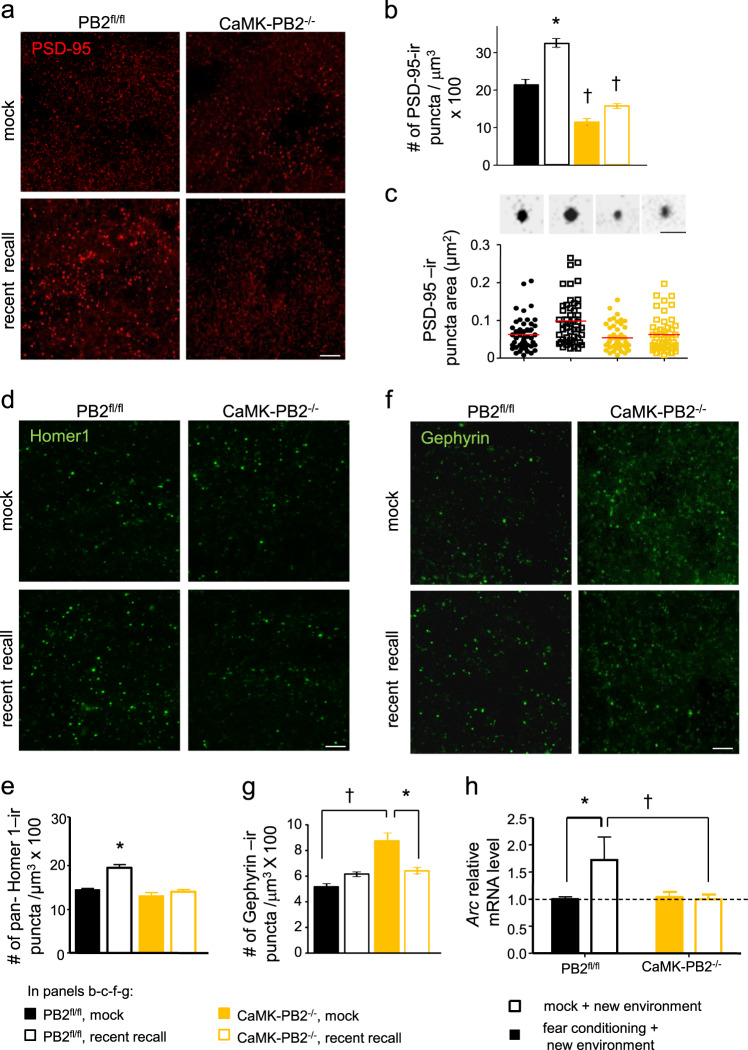
Changes in density of excitatory and inhibitory synapses in the CA1 in vivo upon induction of fear memory and functional role of Plexin-B2 thereof. **a**, **b** Typical examples (**a**) and quantitative analysis (**b**) of the density of excitatory synapses detected as number of anti-PSD-95-immunoreactive puncta of apical dendrites of CA1 pyramidal neurons in CaMK-PB2^−/−^ mice and PB2^fl/fl^ littermates in mock-treated group or at 2 days after fear conditioning (corresponding to recent recall). Data are represented as mean of the number of synapses per unit volume, error bars represent SEM. **c** Scatter-plot representation of the size of PSD-95-immunoreactive puncta on apical dendrites of CA1 pyramidal neurons in CaMK-PB2^−/−^ mice and PB2^fl/fl^ littermates in mock-treated group or at 2 days after fear conditioning (red lines represent the mean); examples of automated unbiased size estimation via software are shown above. Scale bar = 1 µm. **d**, **e** Typical examples (**d**) and quantitative analysis (**e**) of the density of excitatory synapses of apical dendrites of CA1 pyramidal neurons detected via anti-pan-Homer1-immunoreactivity of synaptic puncta on apical dendrites of CA1 pyramidal neurons in the above treatment groups. **f**, **g** Typical examples (**f**) and quantitative analysis (**g**) of the density of inhibitory synapses of apical dendrites of CA1 pyramidal neurons detected as density of anti-gephyrin-immunoreactive puncta on apical dendrites of CA1 pyramidal neurons in the above treatment groups. **a**–**g** *n* = 3 mice per group, at least 3 different sections per mouse, 3 different ROIs per section were analyzed. Two-way ANOVA was employed in **b**, **e** and **g**, followed by Bonferroni post hoc test. In panel **c**, Krusker–Wallis test was employed. **h** Quantitative RT-PCR for *Arc* mRNA expression in the CA1 area of hippocampus of CaMK-PB2^−/−^ mice and PB2^fl/fl^ littermates in mock-treated group or on day 4 after fear conditioning; mice from all groups were subjected to a novel environment one day after recent recall. In panel **h**, ANOVA was employed followed by Holm–Sidak correction for multiple comparisons. *N* = 7 mice per group. * represents *P* *<* 0.05 when mice with fear conditioning and corresponding mock-treated mice were compared and ^†^ represents *P* *<* 0.05 when mutant and corresponding wild-type littermates were tested. Error bars represent S.E.M. Scale bars represent 10 µm unless otherwise indicated above

Not all dendritic protrusions develop into functional synapses. To get an overall picture of changes in synaptic density in specific molecular layers of the hippocampus and to study bonafide synapses, we employed classical neurochemical markers labeling post-synaptic proteins, namely PSD-95, a synaptic scaffold protein highly enriched in excitatory synapses [60], and gephyrin, a synaptic scaffold protein which labels inhibitory synapses [61]. Immunoreactive synapses were quantified as previously described by Pizzo et al. [62]. Negative controls for immunostaining are shown in Supplementary Fig. 5a. Under basal conditions, CaMK-PB2^−/−^ mice showed a significant reduction in the number of anti-PSD-95-immunoreactive (PSD-95-positive) puncta in stratum radiatum of CA1, where apical dendrites make synaptic connections with Schaffer collaterals from CA3 pyramidal neurons, as compared to wild-type controls (Fig. 5a, b). Supplementary Fig. 5b shows the areas chosen for synaptic analysis.

Interestingly, following fear conditioning, control mice showed a significant increase over the basal state in the excitatory synaptic density (PSD-95-positive puncta) at day 2 after fear conditioning in stratum radiatum of CA1 (Fig. 5a, b), i.e., corresponding to the time point of the behavioral analysis of recent recall we reported above. The magnitude was comparable to the increase we observed in spine density after fear conditioning, suggesting excitatory synaptogenesis. In contrast, CaMK-PB2^−/−^ mice did not show any significant alterations over the basal state on day 2 after fear conditioning (Fig. 5a, b). Because spatial learning processes have been reported to be associated with an increase in size of synaptic spines [11, 63, 64], we undertook an unbiased estimation of the size of PSD-95-immunoreactive puncta during recent fear memory induction. In wild-type mice at day 2 after fear conditioning, although a subpopulation of PSD-95-expressing puncta were observed to be of a larger size than the average in mock-treated mice (typical examples in upper panel of Fig. 5c), the net change in size did not reach statistical significance (quantitative summary in lower panel of Fig. 5c; histograms of puncta size given in Supplementary Fig. 5d). The size of PSD-95-immunoreactive puncta was not different between wild-type and CaMK-PB2^−/−^ mice under control conditions or after fear conditioning (Fig. 5c; Supplementary Fig. 5d).

We also undertook a separate set of experiments with an independent marker for excitatory synapses, namely a pan-Homer1 antibody which has been used to label hippocampal post-synaptic structures in several studies and labels a subpopulation of excitatory synapses [65–67]. At day 2 after fear conditioning, wild-type mice showed a significant increase in the density of Homer1-immunoreactive puncta in stratum radiatum of CA1 as compared to mock-treated mice, supporting our observations with anti-PSD-95 immunostaining. Importantly, this increase did not come about in CaMK-PB2^−/−^ mice upon fear conditioning (Fig. 5d, e). Given these observations with two independent synaptic markers, we are inclined to conclude that enhanced density of excitatory synapses in contextual fear conditioning involves Plexin-B2.

We then also addressed how inhibitory synapses are affected by loss of Plexin-B2. In basal state (mock-treated mice), we found an increased density of gephyrin-positive puncta in CaMK-PB2^−/−^ mice as compared to control mice in stratum radiatum of CA1 (Fig. 5f, g). These data suggest that loss of Plexin-B2 led to decreased density of excitatory synapses and an increased density of inhibitory synapses in the stratum radiatum area of the CA1, reflecting a role for semaphorin-plexin-B2 signaling in synaptic maturation and pruning. Moreover, only CaMK-PB2^−/−^ mice, but not control mice, showed a significant decrease in inhibitory synapse density (gephyrin-positive puncta) in stratum radiatum of CA1 at day 2 after fear conditioning. Thus, at the time of recent fear memory recall, the density of gephyrin-positive puncta was comparable between control and CaMK-PB2^−/−^ mice (Fig. 5f, g). Changes in the synaptic density were not due to alteration of neuronal density in the pyramidal CA1 hippocampal area, which was comparable between CaMK-PB2^−/−^ mice and control mice (Supplementary Fig. 5c). These observations suggest that endogenous Sema4C-Plexin-B2 signaling is involved in both basal turnover of mature synapses as well as in synaptic remodeling associated with fear memory recall in the mouse hippocampus.

The most direct way to test the impact of these morphological changes on synaptic function would be via slice electrophysiology. However, while this was beyond the scope of the current study, we analyzed a surrogate molecular parameter for rapid neuronal responsivity to synaptic activity, namely rapid upregulation of *Arc* mRNA upregulation downstream of diverse synapse-to-nucleus messengers [68]. We induced *Arc* mRNA via exposure of mice to a new environment [69–71] that was not associated with any fear memory, and observed that wild-type mice with fear-conditioning showed more than 50% increase in the level of *Arc* induction over mock-treated wild-type mice (Fig. 5h; values normalized to mock-treated mice). In contrast, mock-treated and fear-conditioned CaMK-PB2^−/−^ mice demonstrated comparable levels of *Arc* induction in response to a new environment. These results correlate positively with our morphological observations on enhanced hippocampal dendritic ramifications and synaptic density in wild-type fear-conditioned mice, which are unaltered upon loss of Plexin-B2.

### Sema4C increases dendritic complexity and dendritic spine density in cultured hippocampal neurons

**Fig. 6:**
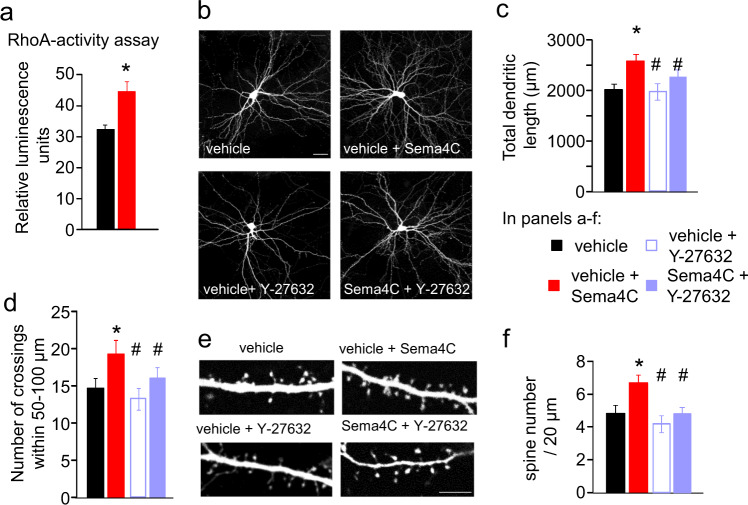
Function of Sema4C in fear and fear memory on dendritic structural remodeling ex vivo. **a** ELISA-based analysis of RhoA activation in cultured mouse hippocampal neurons upon exposure to Sema4C (150 nM for 15 min); *n* = 4 independent culture experiments); Student’s *t*-test, *P < 0.05 as compared to vehicle-treated cultures. **b**–**d** Typical examples (**b**) and quantitative analysis of total dendritic length (**c**) and number of dendritic crossing (**d**; range 50–100µm from the soma) of EGFP-labeled hippocampal neurons from wild-type mice at 10 days in vitro (DIV). **e**, **f** Typical examples (**e**) and quantitative summary (**f**) of morphometric analysis of EGFP-labeled synaptic spines on wild-type hippocampal neurons at 10 DIV upon treatment with vehicle, Sema4C (150 nM), Y-27632 (3.3 µM) or Sema4C (150 nM) + Y-27632 (3.3 µM) for 24 h. In panels **c**, **d**, **f**: one-way ANOVA with post hoc Tukey’s test; *n* = 11–12 neurons per condition, 3 independent culture preparations. *P < 0.05 as compared to vehicle-treated cultures, ^#^*P* < 0.05 as compared to Sema4C-treated neurons. Error bars represent SEM. Scale bars represent 100 µm (**a**, **c**), 20 µm (**g**) and 5 µm (**j**), respectively

To better understand whether Sema4C is capable of modifying neuronal architecture and dendritic spines along the lines, we observed for Plexin-B2 above, we used cultured hippocampal neurons as a model system. To ascertain that Sema4C can signal in our model system, we treated cultured hippocampal neurons with recombinant soluble Sema4C, which we have previously demonstrated to be biologically active [34] and analyzed activation of the Plexin-B-RhoA-ROCK arm of Sema4C-Plexin-B signaling (schematic in Supplementary Fig. 1). Using a pull-down assay, we found that in comparison to vehicle treatment, Sema4C significantly increased the levels of activated endogenous RhoA in hippocampal neurons within 30 min (Fig. 6a). To study neuronal architecture, we transfected hippocampal neurons at 8 DIV with humanized Renilla reniformis green fluorescent protein (hrGFP) and performed our experiments at DIV10, a stage at which, under our experimental conditions, neurons do not extent further their dendritic trees and are mostly stable. We observed that neurons exposed to Sema4C for 24 h showed a significantly higher total dendritic length in comparison to vehicle-treated neurons (Fig. 6b, c). Furthermore, Sema4C significantly increased the complexity of the dendritic tree in hippocampal neurons, as assessed via Sholl analysis (Fig. 6d and Supplementary Fig. 6a). Thus, Sema4C can further foster dendritic growth at a time point when developmental dendritogenesis is already complete.

Both RhoA-ROCK signaling as well as molecular pathways downstream of the receptor tyrosine kinase Met have been implicated in structural modifications by Plexin-B proteins [24, 26, 72]. We therefore tested the involvement of the RhoA-ROCK pathway and the Met signaling pathway in the observed effects of Sema4C on dendritic architecture by pretreating neurons with an inhibitor of ROCK, Y-27632, or an inhibitor of Met, PHA665752, for 30 min prior to the 24 h exposure to Sema4C. At the dose employed, Y-27632 did not per se affect the total dendritic length or complexity of the treated neurons (Fig. 6b, c and Supplementary Fig. 6b, c), but blocked the morphometric effects of Sema4C. Pretreatment with PHA-665752 abolished the trophic effects of Sema4C, but also decreased dendritic length and branching in the absence of Sema4C, thereby rendering it difficult to derive specific contributions towards Sema4C-induced changes (Supplementary Fig. 6b–e).

In parallel, we analyzed the effects of Sema4C exposure on dendritic protrusions and observed that Sema4C treatment led to a significant rise in the density of spines in hippocampal neurons (Fig. 6e, f). Pre-incubation with the ROCK inhibitor Y-27632 abolished the Sema4C-mediated increase in spine density (Fig. 6e, f).

Thus, the Plexin-B2 ligand, Sema4C can bring about acute remodeling of the dendritic architecture in hippocampal neurons ex vivo via RhoA-ROCK signaling, which is consistent in nature with the morphological phenotypes observed in CaMK-PB2^−/−^ mice in vivo.

### An important functional role for RhoA-ROCK signaling downstream of Plexin-B2 in fear memory recall in vivo

To functionally close the circle from RhoA-ROCK-mediated spine and dendritic plasticity to the role that we observed for Plexin-B2 in the recall of fear memory, we sought to address whether RhoA signaling downstream of Plexin-B2 mediates its functions in fear memory in vivo. We have recently described a genetic strategy of specifically mutating Plexin-B2 domains, wherein activation of specific downstream pathways is conditionally hindered. This is based on transgenic rescue of Plexin-B2 expression in global Plexin-B2 knockout mice (PB2^−/−^) [41]. In contrast to PB2^−/−^ mice, which show embryonic or perinatal lethality [24], rescuing endogenous Plexin-B2 expression via the wild-type Plexin-B2 (PB2^−/−^, PB2^wt^) restores survival and enables functional analysis in adult mice [41]. Because deletion of the C-terminal VTDL motif hinders RhoA signaling [20], we have employed the strategy mentioned above to generate mice expressing a Plexin-B2 loss-of-function mutant for the RhoA pathway (PB2^−/−^, PB2-LOF^RhoA;^ for schematic representation see Supplementary Fig. 7a), which are viable and develop normally [41]. We have also previously demonstrated that the expression levels of the wild type Plexin-B2 rescue and RhoA signaling-mutant version of Plexin-B2 are comparable across PB2^−/−^, PB2^wt^ ‘rescue’ mice and PB2^−/−^, PB2-LOF^RhoA^ mice [34].

**Fig. 7:**
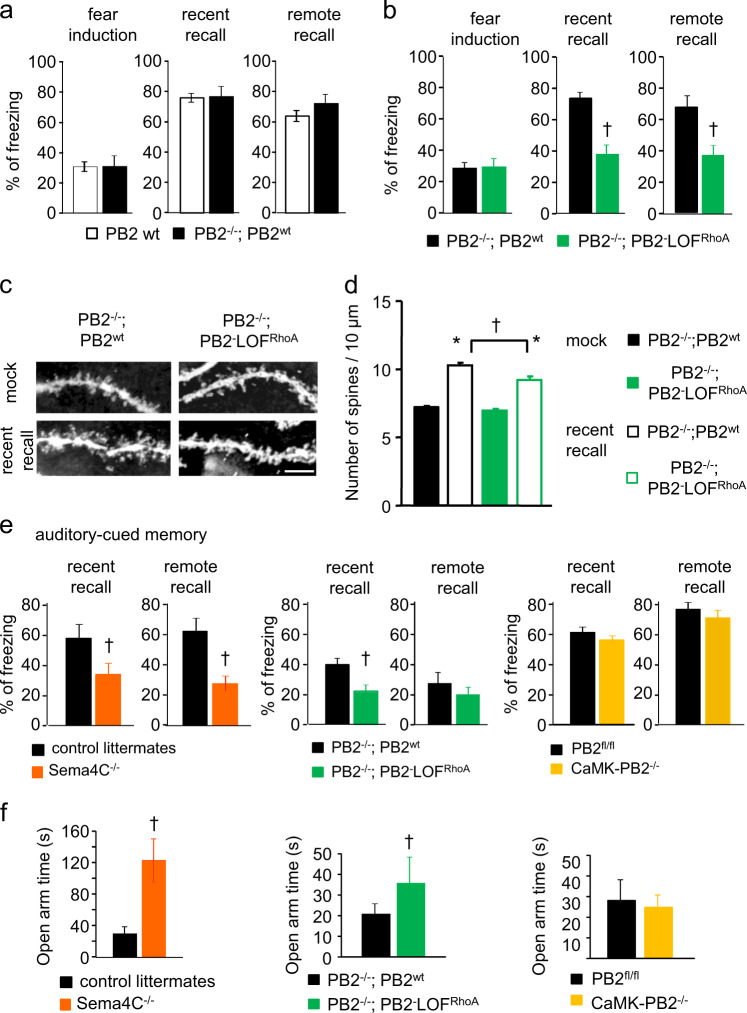
Analysis of the contribution of RhoA-ROCK signaling to the functions of Plexin-B2 in contextual fear memory and role of Sema4C-Plexin-B2 signaling in auditory-cued fear memory and anxiety. **a**, **b** Analysis of fear response and contextual fear memory at recent (day 2) and remote (day 36) stages in control wild-type mice (PB2 wt), transgenic mice with expression of wild-type Plexin-B2 expression in Plexin-B2 global knockout background (PB2^−/−^; PB2^wt^) and transgenic mice with a expression of a Plexin-B2 variant lacking the ability of initiating RhoA-ROCK signaling (PB2^−/−^; PB2-LOF^RhoA^); *n* = 5 mice for wild-type animals, *n* = 5 for PB2^−/−^; PB2^wt^ mice (**a**); *n* = 9 mice for the control group and *n* = 8 for PB2^−/−^; PB2-LOF^RhoA^ mice (**b**). **c**, **d** Typical examples of Golgi-stained dendritic segments with dendritic spines (**c**) and quantitative analysis (**d**) of spine density over apical dendrites of CA1 pyramidal neurons in the treatment groups described above. A minimum of 12 neurons per genotype or treatment from at least 3 mice were analyzed. Two-way ANOVA for repeated measures followed by Bonferroni’s test was performed. *P* < 0.05 indicated by * as compared to the corresponding control groups. **e** Analysis of auditory-cued memory at recent (day 3) and remote stages (day 37) in mice of the designated genotypes; *n* = 7 mice per group for Sema4C^−/−^ and control group; *n* = 9 mice for the control group and *n* = 8 for PB2^−/−^; PB2-LOF^RhoA^ mice; *n* = 8 for CaMK-PB2^−/−^ and control group. **f** Analysis of anxiety behavior in the elevated plus maze (EPM) test evaluating the time spent in the open arm of the EPM apparatus in mice of the designated genotypes; *n* = 8 mice per group for Sema4C^−/−^ and control group; *n* = 9 mice for the control group and *n* = 8 for PB2^−/−^; PB2-LOF^RhoA^ mice; *n* = 8 for CaMK-PB2^−/−^ and control group. In panels **a**, **b**, **e**, **f**, Student’s *t*-test was performed; * represents *P* *<* 0.05 when mice with fear conditioning and corresponding mock-treated mice were compared and ^†^ represents *P* *<* 0.05 when mutant and corresponding wild-type littermates were tested. Error bars represent SEM

The acquisition of fear memory, i.e., freezing response during shock, was comparable between wild-type, PB2^−/−^, PB2^wt^ ‘rescue’ mice and PB2^−/−^, PB2-LOF^RhoA^ mutant mice (Fig. 7a, b), also consistent with observations in CaMK-PB2^−/−^ mice. Importantly, in contrast to CaMK-PB2^−/−^ mice, PB2^−/−^, PB2^wt^ ‘rescue’ mice show comparable freezing responses in both recent- and remote memory recall tasks to wild-type control mice (Fig. 7a), indicating that Plexin-B2 expression and function was fully restored in fear memory circuits neurons using this genetic strategy. In contrast to a rescue with wild-type Plexin-B2, we noted that expression of the Plexin-B2 RhoA signaling-mutant led to impairment of fear memory recall in both early and remote memory contexts (Fig. 7b). Furthermore, this functional change was also accompanied by a reduced density of dendritic spines in CA1 hippocampal neurons in Golgi staining experiments performed at 3 days post-fear conditioning. Thus, PB2^−/−^, PB2-LOF^RhoA^ mice fully recapitulated the functional phenotype in CaMK-PB2^−/−^ mice with respect to contextual fear memory, suggesting that the function of Plexin-B2 in contextual fear memory is mediated by RhoA signaling.

Quantifying spine-like dendritic protrusions in Golgi-stained CA1 neurons revealed that under basal conditions, spine density was comparable in Golgi-stained CA1 neurons in PB2^−/−^, PB2-LOF^RhoA^ mice and control mice (Fig. 7c, d). At time points corresponding to recent fear memory recall, we observed a significant increase in spine density over basal value in both PB2^−/−^, PB2-LOF^RhoA^ mice and control mice (Fig. 7c, d). However, the fear-induced increase in spine density detected in PB2^−/−^, PB2-LOF^RhoA^ mice was significantly lower (*P* < 0.05) than the magnitude seen in control mice (Fig. 7c, d), suggesting a significant contribution of Plexin-B2-mediated RhoA signaling toward spine changes associated with fear memory.

### Analysis of potential roles of Plexin-B2 and Sema4C in auditory-cued fear memory

Finally, we also addressed auditory-cued fear memory in this study, which is believed to involve additional brain areas, such as the amygdaloid nuclei [5]. As shown in Figs. 1 and 2, both Plexin-B2 and Sema4C are expressed in the amygdala. In auditory-cued fear recall tasks after fear conditioning, Sema4C^−/−^ mice as well as PB2^−/−^, PB2-LOF^RhoA^ mice, both of which are constitutive and global mutants, showed impairments in recent memory recall (day 3), in remote in comparison with the corresponding wild type and PB2^−/−^; PB2^wt^ control mice, respectively (Fig. 7e); Sema4C^−/−^ mice also demonstrated a significant decrease in remote memory recall on day 37 (Fig. 7e). In contrast, no phenotypic differences were between CaMK-PB2^−/−^ mice and their corresponding PB2^fl/fl^ littermates with respect to auditory-cued fear memory at recent or remote stages (Fig. 7e). As expected, no phenotypic differences were observed between between PB2^−/−^, PB2^wt^ mice and PB2^wt^ control mice (Supplementary Fig. 7b). Moreover, constitutive PB1^−/−^ mice also did not differ from their control littermates (Supplementary Fig. 7c).

To further understand the role of Sema4C-Plexin-B2 signaling in the amygdala, we tested anxiety behavior using elevated plus maze (EPM) test, which is linked to amygdala function [73]. We observed that PB2^−/−^; PB2-LOF^RhoA^ mice as well as Sema4C^−/−^ mice spent significantly more time in the open arms of EPM as compared to their respective controls (Fig. 7f), indicating a strong impairment of the open space-induced anxiety. In contrast, CaMK-PB2^−/−^ mice showed comparable levels of anxiety in the EPM test as their control littermates (Fig. 7f panel at the right), consistent with behavior in auditory-cued memory recall.

## Discussion

Aversive experiences alter neuronal function in specific neural pathways, thereby triggering formation of aversive memories, which change behavior. Fear disorders can result when emotional responses persist in the absence of danger or aversive predictors, and form the core of several psychiatric disorders, such as post-traumatic stress disorder and phobias. Recently, there has been tremendous progress in our understanding of the identity of neural circuits of fear and fear memories [74, 75]. However, the basic molecular and cellular underpinnings of memory formation and maintenance in fear circuits remain unclear and are of considerable translational interest. On this background, the present study now reports that a semaphorin-plexin ligand-receptor pair, namely Sema4C-Plexin-B2, is an important regulator of fear memory formation in forebrain circuits and its long-term maintenance, which is linked to its ability to structurally reshape dendritic arborizations and glutamatergic and GABAergic synapses on excitatory neurons. This study also reports the unexpected finding that RhoA-dependent signaling downstream of Plexin-B2 activation is a positive force in activity-dependent generation of new spines in the context of fear memory.

Semaphorins and plexins are evolutionarily conserved sculptors of organismal development, which are now being appreciated more and more in the context of key functions in adult life [30, 33]. This study complements our recent report describing a novel role for Sema4C-Plexin-B2 RhoA signaling in adult peripheral sensory neurons, where it modulates inflammatory nociceptive hypersensitivity during adult life [34], in underlining the emerging principle of reactivation of developmental cues in specific instances of adult pathophysiology.

Because fear memory relies on interactions between hippocampal-, amygdaloid- and prefrontal cortical circuits in the forebrain [7, 8], these constituted the primary focus of our analyses. The hippocampus plays a central role in learning and memory, and plasticity of synapses within different hippocampal sub-regional circuits of the dentate gyrus–CA3–CA1 regions is known to underlie memory formation via potentiation of synaptic strength [76]. Our analyses suggest that both the receptor Plexin-B2 as well as the ligand Sema4C is expressed in all hippocampal sectors, opening the possibility for autocrine as well as paracrine signaling. Enhanced CA3 to CA1 paracrine signaling is likely in fear memory since a strong aversive fear-inducing stimulus was observed to enhance the expression of the ligand in CA3 pyramidal neurons and the receptor in CA1 pyramidal neurons. Plexin-B2 expression was also increased in dentate granule cells in fear memory paradigms, which may suggest enhanced signaling from incoming cortical afferents, consistent with the widespread cortical expression of Sema4C we observed. Owing to technical hindrances with antibodies in immunohistochemical analyses, we utilized LacZ reporter lines to address expression and regulation of Plexin-B2 and Sema4C expression, particularly because we have previously characterized these reporter mice in detail and demonstrated that they faithfully recapitulate the patterns of expression as well as quantitatively (although not linearly) reproduce the regulation of the *Plxnb2* and *Sema4c* gene loci, respectively [34]. Nevertheless, it was important to comfirm our findings with mRNA in situ hybridization as well as western blotting, which largely confirmed our results from the LacZ reporter lines.

Therefore, a major thrust of our experiments was placed on functional experiments testing the significance of Sema4C-Plexin-B2 signaling towards fear-related behaviors. The generation and use of CaMK-PB2^−/−^ mice enabled us to test the role of Plexin-B2 in adult neurons free of developmental caveats and in the forebrain. Sema4C^−/−^ mice and PB2^−/−^, PB2-LOF^RhoA^ mice that were employed here provided important insights into contributions of the ligand and the signaling pathway, respectively, but represent global, constitutive knockouts. The commonalities and differences in the phenotypic manifestation of fear behavior in these diverse lines therefore also enabled distilling some interesting insights into contextual versus cued fear memory. Taking the commonalties into account, the most important and consistent feature which emerged is that Plexin-B2-RhoA signaling in forebrain excitatory neurons makes a significant contribution to the expression of both recent and remote recall of contextual memory. The role of the hippocampus in recent contextual memory recall is paramount, but the ACC has been postulated to be more important for storage and retrieval of remote memories [77]. Recent studies support the “multiple trace theory”, which suggests that the hippocampal memory trace is not replaced by the cortical one (as previously thought), but rather both memories are in constant interplay [59, 78–80]. Reporter mouse analyses suggested here that Plexin-B1 and Sema4C are expressed in both hippocampus and the ACC, and the ligand is upregulated in the ACC by strongly aversive stimuli that establish long-term fear memories.

A second clear observation was that auditory-cued memory was impaired in Sema4C^−/−^ and PB2^−/−^, PB2-LOF^RhoA^ mouse lines, but not in CaMK-PB2^−/−^ mice. Amygdaloid circuits are of key importance in auditory-cued fear memory [3, 5], but the lack of phenotypic changes in CaMK-PB2^−/−^ mice did not stem from the lack of Cre recombination in the amygdala in the CaMKCreER^T2^ line employed here [38]. Rather, this difference may stem from differential targeting of excitatory and inhibitory neurons; while Sema4C^−/−^ and PB2^−/−^, PB2-LOF^RhoA^ mouse lines involve constitutive and global recombination (i.e., in cell types expressing the respective genes), the CaMK-PB2^−/−^ mice we generated stem from the CaMKCreER^T2^ mouse line, which is specific to excitatory neurons [38]. Inhibitory circuits involving GABAergic neurons are reported to play a critical role in auditory fear memory in diverse centers [81, 82], including the auditory cortex [83] and the amygdala [84]. In particular, the CeA is well characterized by its inhibitory microcircuits [5, 85, 86], which gate output to control the level of conditioned freezing. These inhibitory neurons are not affected in the CaMKCreER^T2^ line we used [38], suggesting that amygdala-dependent responses involving inhibitory neurons remain intact in CaMK-PB2^−/−^ mice. Consistent with the above, CaMK-PB2^−/−^ mice demonstrated no differences with respect to another key behavior linked to amygdaloid output, namely anxiety in open space. In contrast, both Sema4C^−/−^ and PB2^−/−^, PB2-LOF^RhoA^ mouse lines showed impairment of behaviors related to both auditory-cued fear memory and anxiety, suggesting a role for Sema4C acting via Plexin-B2 in inhibitory amygdaloid circuits. It is also plausible that other brain regions not targeted in CaMK-PB2^−/−^ mice (i.e., beyond the forebrain), but affected in the constitutive and global Sema4C^−/−^ and PB2^−/−^, PB2-LOF^RhoA^ mutants, come into play in regulating auditory-cued fear memory and anxiety. Finally, it cannot be ruled out that potential developmental defects in constitutive and global Sema4C^−/−^ and PB2^−/−^, PB2-LOF^RhoA^ mutants, that are lacking in CaMK-PB2^−/−^ mice with adult-onset Plexin-B2 deletion contribute to these phenotypic differences. Although we observed the dendritic architecture in the hippocampal CA1 region to be normal in Sema4C^−/−^ and PB2^−/−^, PB2-LOF^RhoA^ mice, it cannot be ruled out that other regions involved in auditory fear memory, such as the amygdala, are affected.

Given the consistent phenotypic changes in contextual memory paradigms, we focused our mechanistic analyses on the hippocampus. Here, we observed that fear conditioning with a strong aversive stimulus markedly enhanced dendritic arborizations as well as the spine density on apical and basal dendrites of CA1 neurons, and the magnitude of these structural changes was consistent with previous literature on fear memory paradigms [57, 58]. Although dendritic length has not been widely studied before, we also observed a major increase in overall dendritic length upon fear conditioning, which can be conceivably linked to an increase in dendritic arborizations. This overall increase in dendritic growth as well as dendritic spines across the CA1 may appear surprising given that contextual fear memory recall is functionally linked to specific and discrete sets of neurons, i.e., engrams, in the hippocampus [87, 88]. It is conceivable that although only ensemble or engram neurons are specifically recruited, they modulate activity and consequently structure of several connected cells in the local environment, and that Plexin-B2-Sema4C signaling plays a role in this local amplification of plasticity. Indeed, large-scale structural changes that have been described in several paradigms of plasticity, including stress [89, 90]. Nevertheless, it remains a challenge for future studies to understand how activity of a small set of engram neurons can be linked to large-scale structural reorganization in the context of fear memory.

Importantly, we observed that Sema4C-Plexin-B2 signaling establishes a vital mechanistic link between fear memories and structural plasticity of neurons in hippocampal CA1 neurons. Previous studies have linked semaphorins to dendritogenesis in the developmental context [28], with both positive modulation (e.g., with Sema4D [25]) and negative modulation (such as with Sema3F [36]) being reported. However, modulation of adult dendritic remodeling has not been addressed. Here, in keeping with our interest in plasticity in adult circuits of fear, we tested fully matured hippocampal neurons in vitro or in vivo over recent and remote memory stages and found that an adult-onset loss of Plexin-B2 in hippocampal pyramidal neurons impaired the ability of both basal and apical dendrites of CA1 neurons to show increased ramification over the development of recent memory. Consistent with the above, supplementing Sema4C increased dendritic length and ramifications in cultured mature hippocampal neurons, which could be attributed to signaling via RhoA-dependent ROCK signaling. Although RhoA signaling is classically associated with disassembly of dendritic spines, previous studies, including ours, have demonstrated that the net impact of RhoA-dependent pathways on dendritic morphology is highly context-dependent [26]. Moreover, the Met kinase, which is activated upon Sema4C-Plexin-B2 signaling [91], but also recruited by other trophic cues, such as hepatocyte growth factor (HGF), was observed to contribute to dendritic remodeling of hippocampal neurons, consistent with previous reports in HGF-mediated changes in cortical neurons [92].

Activity-dependent plasticity of synaptic spines plays a central role in consolidation of spatial memories in the hippocampus [14, 15]. Along these lines, a salient and relevant observation of this study was that Plexin-B2-dependent signaling is important for adult plasticity of mature synapses. Interestingly, semaphorins have been linked to synaptogenesis in a few studies previously, but in a developmental context. In particular, Sema5A has been reported to negatively regulate synaptogenesis in early, developmentally born, hippocampal dentate granule cells via Plexin-A2 [93] and another class 4 semaphorin, Sema4D, was reported to promote the development of GABAergic synapses in the hippocampus [25] and to enhance recruitment of pre- and post-synaptic molecules in hippocampal neurons in vitro by acting via Plexin-B1 [94]. In our study, we observed that a loss of Plexin-B2 from mature hippocampal pyramidal neurons decreased the number of PSD-95-labeled excitatory connections and enhanced the density of gephyrin-labeled inhibitory connections in the hippocampal stratum radiatum of naive mice, thereby indicating that the basal turnover of synapses in mature CA1 neurons is tonically regulated by Plexin-B2 signaling. The tonic expression of both receptor and ligand in the hippocampus would support this notion. Taken together, the results from Kuzirian et al. (2013) [94] on Sema4D, which is a high affinity ligand for Plexin-B1, and our data on Plexin-B2 and Sema4C, suggest that Plexin-B1 and Plexin-B2 (and their high affinity ligands Sema4D and Sema4C, respectively) have complementary functional roles in the maintenance of inhibitory and excitatory synapses in the adult CA1, respectively; furthermore, these studies collectively suggest that Plexin-B signaling pathways regulate the overall balance of excitatory and inhibitory connectivity on to hippocampal pyramidal neurons. Importantly, a loss of Plexin-B2 expression also abrogated fear-induced increase in the density of glutamatergic synapses, further providing a structural correlate for the observed functional impairments in fear memory. Sema4C-Plexin-B2-induced increase in synaptic connectivity and dendritic growth and complexity would be expected to enhance the ability of apical and basal dendrites of CA1 neurons to receive and integrate signals from other hippocampal sectors and cortical inputs during the establishment and recall of fear memory. Although we do not have electrophysiological evidence to pinpoint this, our hypothesis was supported by analyses of activity-induced *Arc* mRNA upregulation as a molecular indicator for rapid neuronal responsivity to synaptic activity [68] (in addition to being a marker for activity engrams in fear memory [88]) and itself being a modulator of synaptic strength and neuronal responsivity in turn [68]. *Arc* mRNA is rapidly upregulated in a new environment downstream of diverse synapse-to-nucleus messengers [68, 71, 95], and we observed that fear-conditioned mice showed a larger upregulation of *Arc* towards a new environment than mock-treated mice, supporting our morphological observations of higher synaptic connectivity in hippocampal neurons upon fear conditioning. In contrast, mock-treated CaMK-PB2^−/−^ mice demonstrated normal *Arc* induction in response to a new environment and this did not increase further in magnitude in fear-conditioned CaMK-PB2^−/−^ mice. Thus, these results, obtained with an indirect molecular indicator of synapse-to-nucleus signaling and neuronal responsivity, are consistent with a role for Plexin-B2 signaling altered synaptic and dendritic function in fear-conditioned mice.

Finally, the role of RhoA signaling in these novel functions of Plexin-B2 in synaptic remodeling during fear memory formation deserves some discussion. Our experiments with pharmacological inhibitors in vitro as well as with PB2^−/−^, PB2-LOF^RhoA^ mice in fear memory paradigms in vivo indicate that RhoA signaling is important in enhancing spine density via Sema4C-Plexin-B2 signaling. According to the conventional viewpoint, the RhoGTPase Rac1 stabilizes dendritic spines via LIM Kinase-dependent mechanisms, whereas RhoA is believed to bring about a rapid disassembly of spines via actin-myosin coupling [96]. This notion, has, however, been challenged in recent studies. An elegant study involving dynamic imaging of RhoGTPases addressed the role of RhoA in single dendritic spines undergoing structural plasticity in CA1 neurons during long-term potentiation (LTP) in vivo [97]. Particularly, it reported that RhoA was rapidly activated in the stimulated spines undergoing structural plasticity in a CaMKII-dependent manner and RhoA activation spread into the dendrites over several micrometers and invaded the neighboring spines [97]. Inhibition of the RhoA-ROCK pathway inhibited initial spine growth, indicating that RhoA is involved in the generation of new spines [97]. Our study supports this notion and now reports its significance in the context of synaptic remodeling in fear memory.

Taken together, the present study identifies the Sema4C–Plexin-B2 ligand-receptor pair as an important part of the mechanistic underpinnings of long-term storage and retrieval of fear memories. Because chronic fear has been linked to emotional disorders, this pathway may represent a new avenue for interventions to be harnessed for clinical usage.

## Supplementary information

supplementary figure legends

Supplementary Figure 1

Supplementary Figure 2

Supplementary Figure 3

Supplementary figure 4

Supplementary Figure 5

Supplementary Figure 6

Supplementary Figure 7
