# Supplementary material for: The impact of Semaphorin 4C/Plexin-B2 signaling on fear memory via remodeling of neuronal and synaptic morphology
Source: Mol Psychiatry. 2019 Aug 23;26(4):1376–98. doi: 10.1038/s41380-019-0491-4 (PMC7985029; doi:10.1038/s41380-019-0491-4)
Supplement: Supplementary file 1 — supplementary figure legends [file 41380_2019_491_MOESM1_ESM.docx]

**Simonetti, Paldy et al. Supplementary information**

**Figure S1. Schematic representation of the Sema4C/Plexin-B2 signaling pathway tested in this study (a) as well as of the fear memory testing protocols employed (b).**

**Figure S2. Expression patterns of Plexin-B2 and Sema4C in brain regions involved in fear and fear memory.**  (a) Low magnification images of β-galactosidase expression in mice expressing the LacZ gene in the *Plxnb2* locus (Plexin-B2-LacZ mice; left images) or in the *Sema4c* locus (Sema4C-LacZ mice; right images) across the anterior cingulate cortex (rACC; upper), the amygdale (middle) and the hippocampus (lower). Scale bars represent 200 µm.

(b) Example of areas chosen for separate analysis of LacZ staining signal intensity in the high and low expression level zone (medial and lateral areas, respectively; indicated by arrow and arrowhead, respectively); (c) example of LacZ signal intensity detected in single areas in mock-treated mice (dark bars) or after fear conditioning (blue bars) in the medial zone of rACC (filled bars) or lateral zone of rACC (slashed bars).

**Figure S3. Controls for mRNA *in situ hybridization* (ISH) experiments using the RNAscope technique.**

(a) Negative controls for *in situ hybridization* experiments using RNAscope ISH technique. To test the specificity of *Plxnb2* and *Sema4c* probes, we hybridized brain sections from CaMK-PB2^-/-^ mice (left) and *Sema4c* global knock out mice (right), respectively. Scale bar = 5 µm (b-c) Typical examples of *Plxnb2* (b) and *Sema4c* (c) mRNA expression across the CA1 area of hippocampus (left panels), rACC (middle panels) and in the amygdala (right panels) observed via RNAScope ISH in naïve mice (basal) and mice subjected to fear conditioning. Scale bar = 10µm

**Figure S4. Analysis of spine density in hippocampal CA1 neuronal dendrites.**

(a) Typical example of a Golgi-stained CA1 hippocampal neuron before (left) and after (right) computational processing using Simple Neurite Tracer plugin to create a representative traced image of the neuron in binary mode. (b) Quantitative analysis of spine density in basal (left) and apical (right) dendritic segments in CA1 pyramidal neurons in naïve mice and at the time of remote contextual memory recall (36 days) in PB2^fl/fl^ and CaMK-PB2^-/-^ mice. Two-way ANOVA followed by Bonferroni ’s test was performed. In all panels, a minimum of 12 neurons from at least 2 independent preparations were analyzed. * represents *P* < 0.05 as compared to the corresponding control groups and ^†^ represents *P* < 0.05 as compared to naïve mice (i.e. basal state). Error bars represent S.E.M. Scale bars represent 20 µm.

**Figure S5. Controls for immunohistochemistry experiments and analysis of neuron density in the CA1 pyramidal layer.**

(a) Negative controls for immunofluorescence staining of synapses in the stratum radians in the absence of the respective primary antibodies. Scale bars indicate 5 µm. (b) Representative images showing hippocampal areas considered for the analysis of synaptic density experiments. (c) Quantitative analysis of the density of CA1 pyramidal neurons, detected as anti-NeuN immunoreactive cells, in naïve mice and at the time of recent contextual memory recall (2 days) in PB2^fl/fl^ and CaMK-PB2^-/-^ mice; n = 3 mice/group, at least 3 different sections/ mouse, 3 different ROIs/section were analyzed. Two-way ANOVA was performed followed by Bonferroni post-hoc test. Error bars represent S.E.M. (d) Histograms of anti-PSD-95- immunoreactive puncta size of PB2^fl/fl^ (left) or CaMK-PB2^-/-^ (right) mice after mock treatment (filled bars) or fear conditioning (empty bars).

**Figure S6. Analysis of impact of inhibition of RhoA-ROCK signaling on Plexin-B2-mediated effects on dendritic complexity of hippocampal neurons.**

(a) Cumulative frequency plot of Sholl analysis of the number of dendritic crossings in EGFP-labeled CA1 hippocampal neurons at 10 DIV upon treatment with vehicle or Sema4C (150 nM) in the presence or absence of Y27632 (3.3 µM). (b-e) Typical examples (b) and quantitative summary of changes in dendritic morphology, analyzed as total dendritic length (c), cumulative frequency plot of Sholl analysis (d) and plot of the average number of dendritic crossings (e) in EGFP-labeled CA1 hippocampal neurons at 10 DIV upon treatment with vehicle or Sema4C (150 nM) in the presence or absence of PHA 665752 (2.5 µM); ANOVA followed by Bonferroni’s test. The Sema4C- and vehicle group data shown in panel S6c and S6e are the same as shown in main figures 6c and 6d respectively. T* P < 0.05 as compared to corresponding control (vehicle treated neurons) # P< 0.05 as compared to Sema4C treated neurons; n= 11-12 neurons per condition, 3 independent culture preparations. Error bars represent S.E.M. Scale bar represents 20 μm in panel b

**Figure S7.** **Schematic representation of the allelic series of transgenic Plexin-B2 mice carrying specific mutation in the intracellular domain resulting in loss-of-function (LOF) of the RhoA pathway and analysis of auditory-cued memory across diverse test groups of mice.**

(a) In mice globally lacking *Plxnb2*, mice expressing either transgenic wild-type Plexin-B2 (PB2^-/-^; PB2^wt^) or a transgenic Plexin-B2 variant lacking the ability of initiating RhoA-ROCK signaling (PB2^-/-^; PB2-LOF^RhoA^) were generated. (b, c) Analysis of auditory-cued memory at recent (day 3) and remote stages (day 37) in mice of the designated genotypes; n = 7-9 mice/group. Student´s t-test was performed. P<0.05 indicated by * as compared to the corresponding control groups.
