# Supplementary figures and images for: The impact of Semaphorin 4C/Plexin-B2 signaling on fear memory via remodeling of neuronal and synaptic morphology
Source: Mol Psychiatry. 2019 Aug 23;26(4):1376–98. doi: 10.1038/s41380-019-0491-4 (PMC7985029; doi:10.1038/s41380-019-0491-4)

a

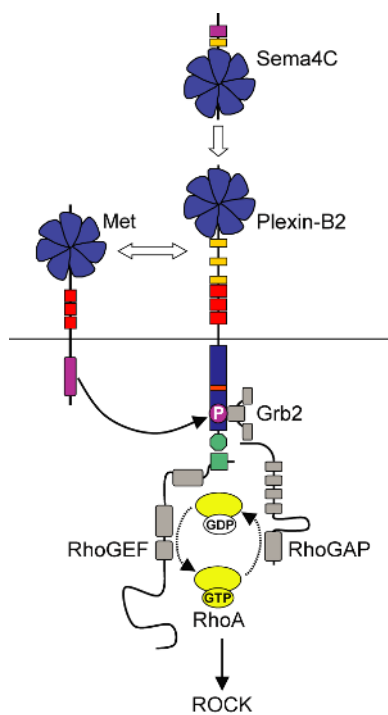

b

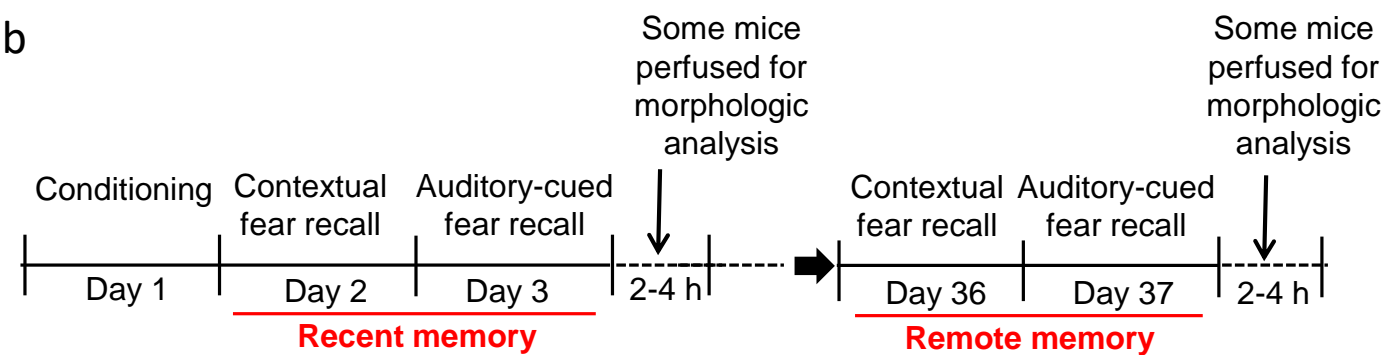

Supplement: Supplementary file 2 — Supplementary Figure 1 [file 41380_2019_491_MOESM2_ESM.pdf]

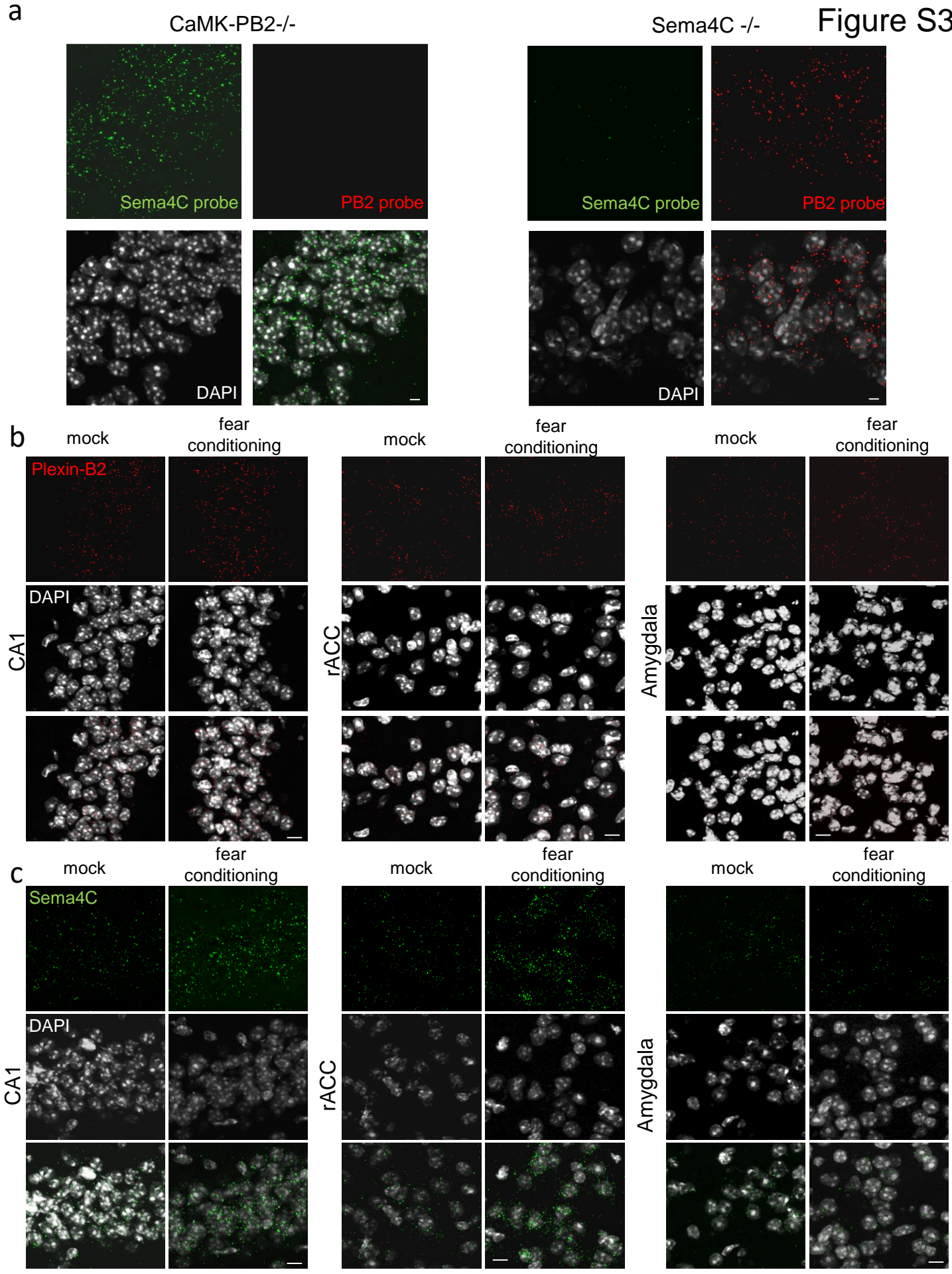

Supplement: Supplementary file 4 — Supplementary Figure 3 [file 41380_2019_491_MOESM4_ESM.pdf]

a

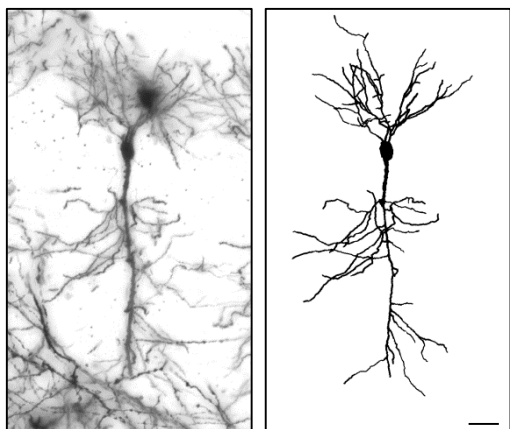

b

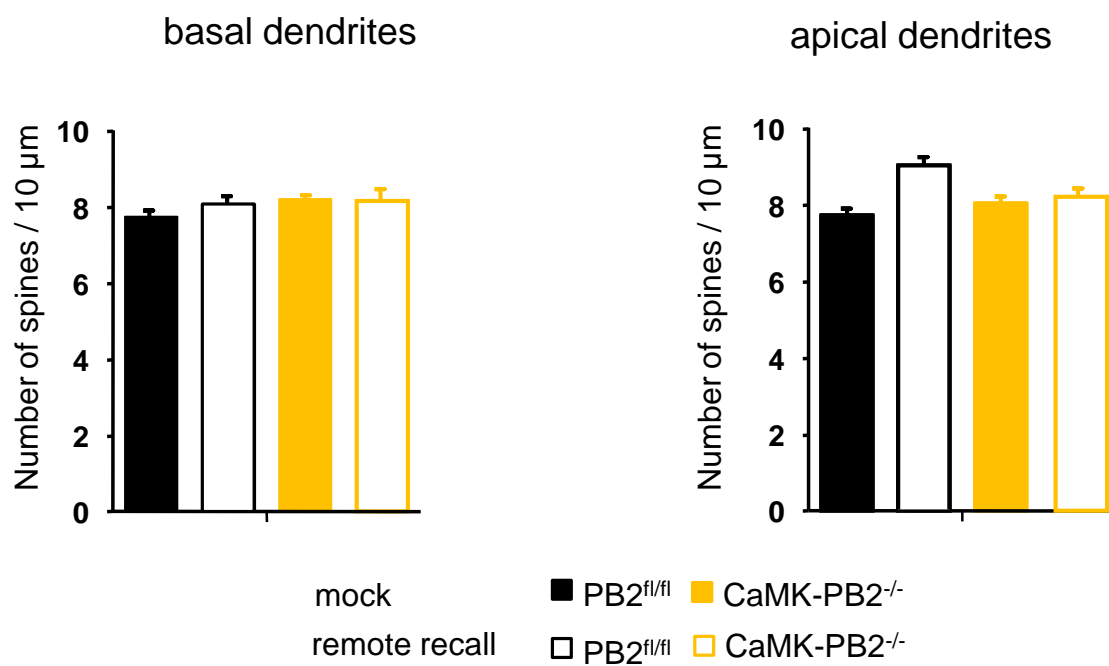

Supplement: Supplementary file 5 — Supplementary figure 4 [file 41380_2019_491_MOESM5_ESM.pdf]

a

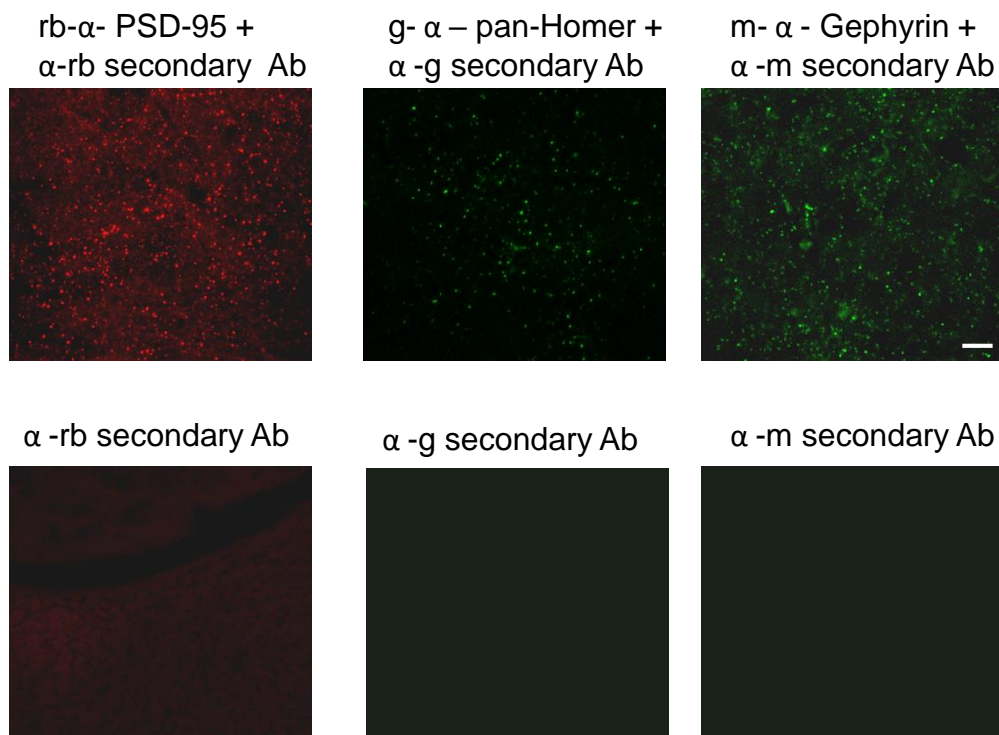

b

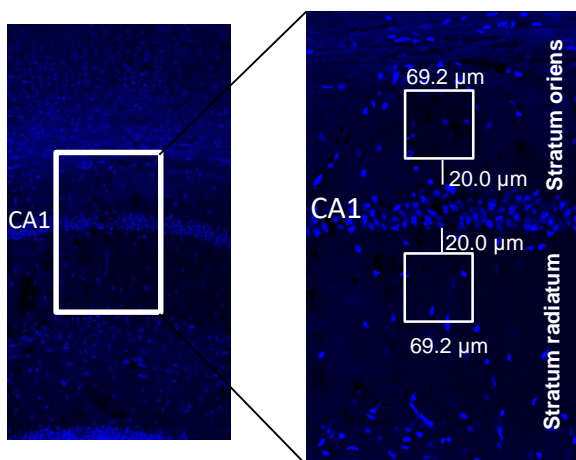

c

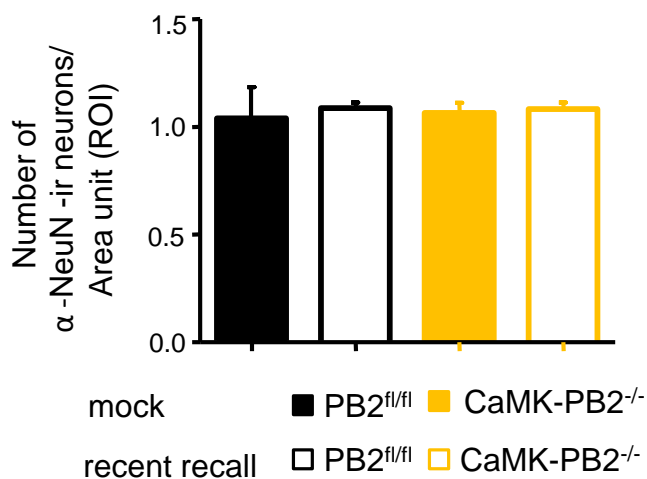

d

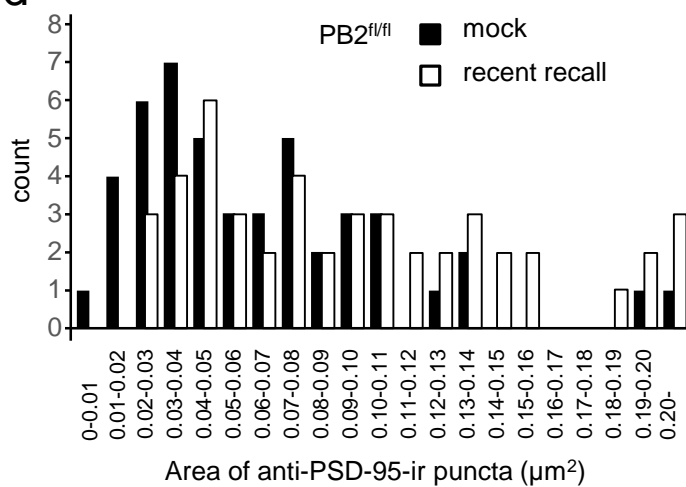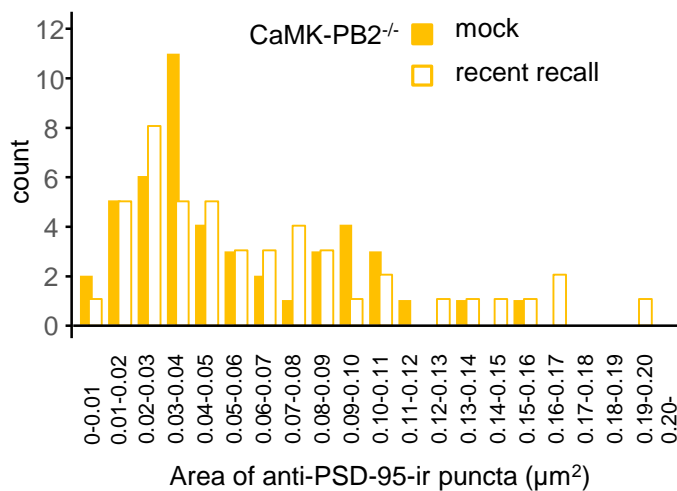

Supplement: Supplementary file 6 — Supplementary Figure 5 [file 41380_2019_491_MOESM6_ESM.pdf]

a

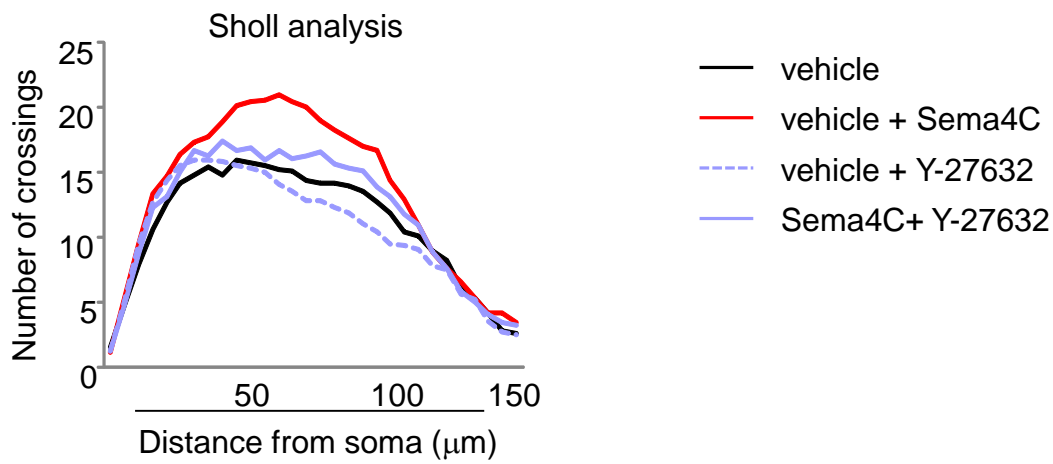

b

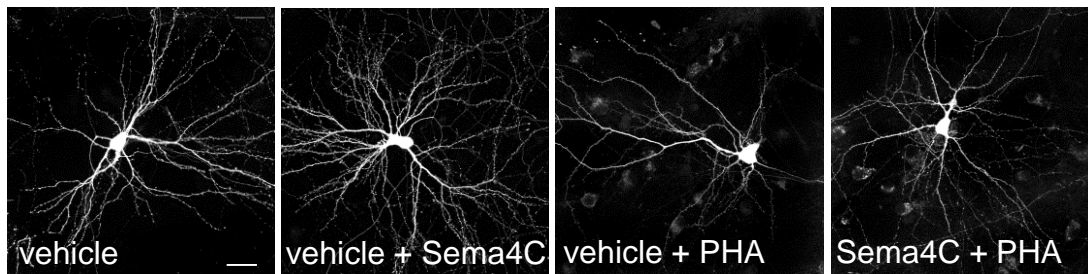

c

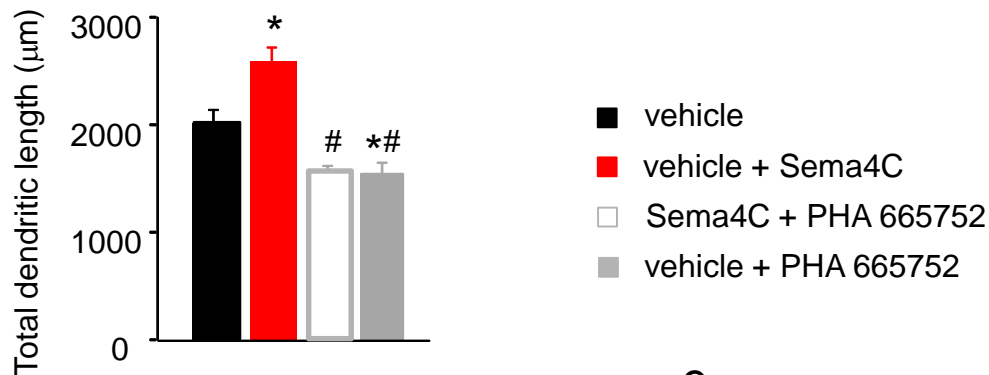

d

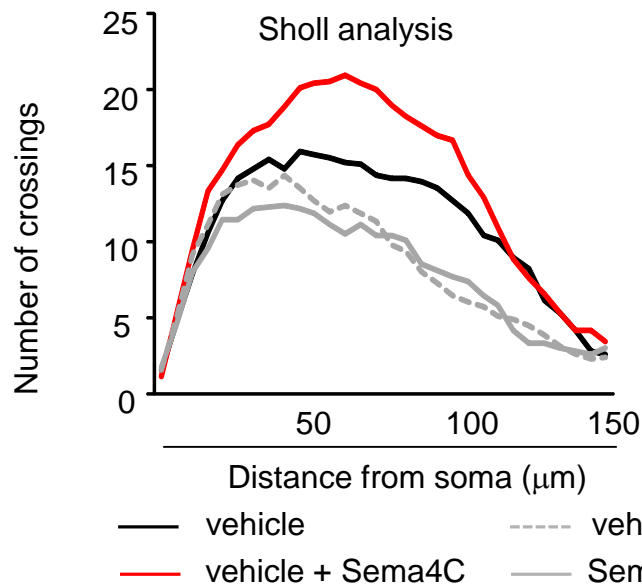

e

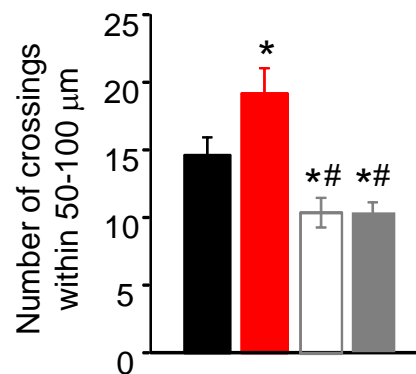

Supplement: Supplementary file 7 — Supplementary Figure 6 [file 41380_2019_491_MOESM7_ESM.pdf]

a

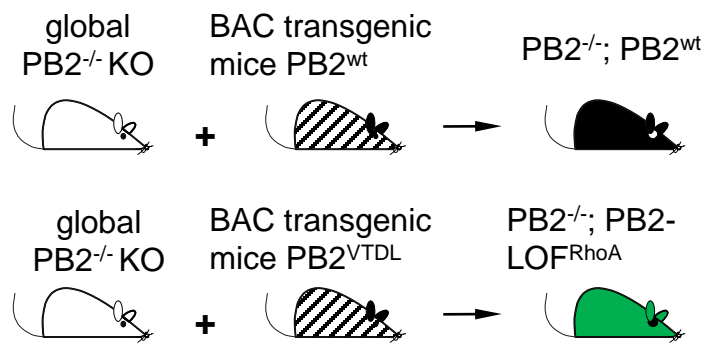

b

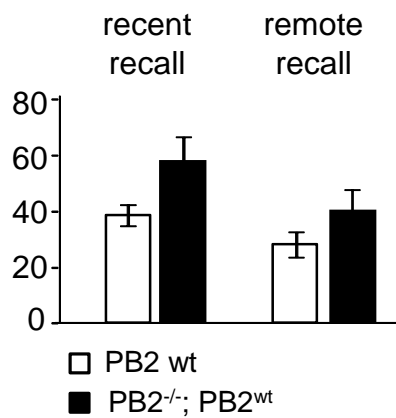

c

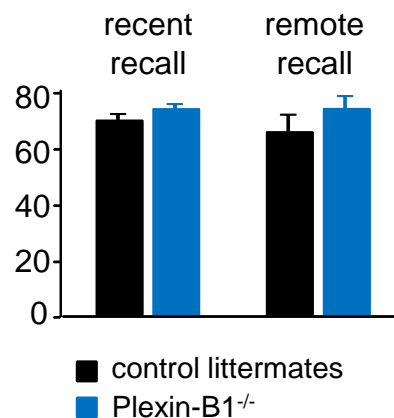

Supplement: Supplementary file 8 — Supplementary Figure 7 [file 41380_2019_491_MOESM8_ESM.pdf]
