# Supplementary material for: The impact of Semaphorin 4C/Plexin-B2 signaling on fear memory via remodeling of neuronal and synaptic morphology
Source: Mol Psychiatry. 2019 Aug 23;26(4):1376–98. doi: 10.1038/s41380-019-0491-4 (PMC7985029; doi:10.1038/s41380-019-0491-4)
Supplement: Supplementary file 3 — Supplementary Figure 2 [file 41380_2019_491_MOESM3_ESM.pdf]

Figure S2

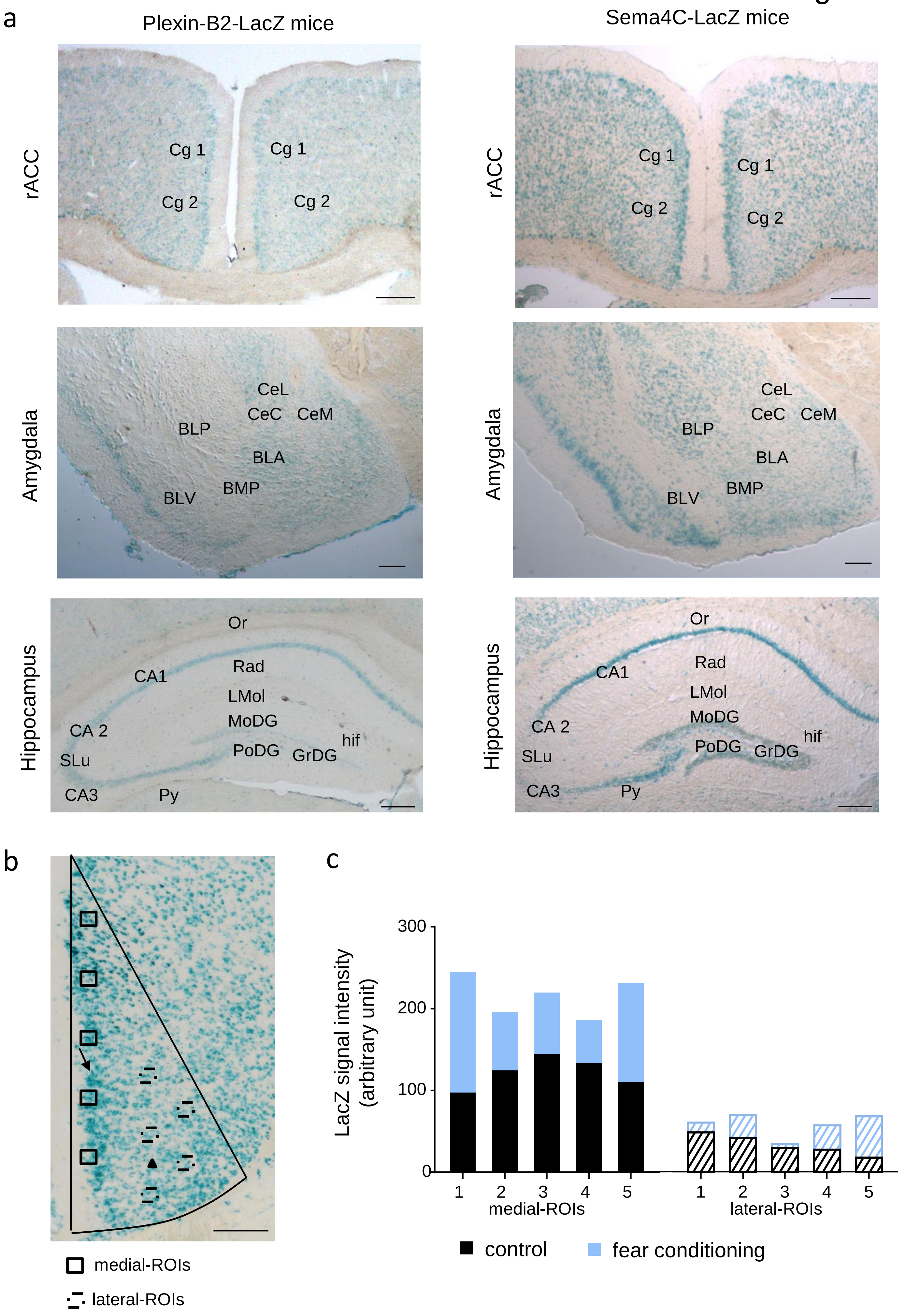

a
Plexin-B2-LacZ mice
Sema4C-LacZ mice
rACC
Cg 1
Cg 2
Amygdala
CeL
CeC
CeM
BLP
BLA
BMP
BLV
Hippocampus
Or
Rad
CA1
LMol
MoDG
CA 2
SLu
PoDG
GrDG
hif
CA3
Py
b
medial-ROIs
lateral-ROIs
c
LacZ signal intensity (arbitrary unit)
300
200
100
0
1
2
3
4
5
medial-ROIs
lateral-ROIs
control
fear conditioning
